# TiO_6_ as a Compensatory Structural Unit in Co‐Free, High‐Ni Layered Oxide for Durable Lithium‐Ion Batteries

**DOI:** 10.1002/advs.77138

**Published:** 2026-08-24

**Authors:** Bixian Ying, Zhenjie Teng, Jun Wang, Honghong Tian, Charlotte von Petersdorff‐Campen, Rommel T. Tolla, Iuliia Mikulska, Linus Voigt, Sascha Nowak, Verena Naber, Alexander Schökel, Yuanming Liu, Michael Merz, Peter Nagel, Stefan Schuppler, Katja Frenzel, Adrian Jonas, Lena Mathies, Martin Winter, Karin Kleiner

**Affiliations:** ^1^ MEET Battery Research Center University of Muenster Muenster Germany; ^2^ School of Innovation and Entrepreneurship Southern University of Science and Technology Shenzhen China; ^3^ Diamond Light Source Ltd Harwell Science & Innovation Campus Oxfordshire UK; ^4^ Deutsches Elektronen‐Synchrotron DESY Hamburg Germany; ^5^ Institute of Materials Research Tsinghua Shenzhen International Graduate School Tsinghua University Shenzhen China; ^6^ Karlsruhe Nano Micro Facility (KNMFi) Karlsruhe Institute of Technology (KIT) Eggenstein‐Leopoldshafen Germany; ^7^ Institute For Quantum Materials and Technologies Karlsruhe Institute of Technology Karlsruhe Germany; ^8^ Physikalisch‐Technische Bundesanstalt (PTB) Berlin Germany; ^9^ Helmholtz‐Institute Münster IMD‐4 Forschungszentrum Jülich GmbH Münster Germany

**Keywords:** compensatory structural unit, elimination of Jahn–Teller distortion, high‐Ni layered oxides, lithium‐ion batteries, phase transition mitigation, Ti substitution

## Abstract

High‐Ni layered oxides are among the most promising cathode materials for lithium‐ion batteries (LIBs) due to their high capacity. However, the reliance on Co, a scarce, expensive, and geographically constrained element, poses challenges to sustainability and large‐scale deployment. In this study, Ti is investigated as a cost‐effective and earth‐abundant substitute for Co in LiNi_1‐x_Ti_x_O_2_ (LNTO, *x* = 0.07 and 0.14), synthesized via a scalable co‐precipitation method. EXAFS analyses indicate that no obvious Jahn–Teller distortion is observed in LNTO‐1, while DFT calculations suggest that Ti substitution modulates the Ni─O electronic interaction and helps reduce the driving force for cooperative Jahn–Teller distortion within the interconnected NiO_6_ framework. In addition, it suppresses the detrimental H2–H3 phase transition and reduces internal mechanical stress during cycling. EXAFS and NEXAFS analyses further reveal that TiO_6_ octahedra act as compensatory structural units that accommodate lattice strain in both bulk and surface regions, thereby stabilizing the rhombohedral framework. As a result, the optimized LNTO‐1 (*x* = 0.07) delivers a high initial discharge capacity of 197.5 mAh g^−^^1^ at 0.05 C and retains 80% capacity after 431 cycles at 0.5 C. This work provides strategic insights for the design of sustainable, high‐performance cobalt‐free Ni‐rich cathodes for next‐generation LIBs.

## Graphical abstract

Ti substitution stabilizes Co‐free high‐Ni layered oxides through both structural and electronic effects. TiO_6_ octahedra act as compensatory structural units that help interrupt cooperative Jahn–Teller distortion and suppress detrimental phase transitions, while the electron‐withdrawing inductive effect of Ti stabilizes the oxygen lattice. As a result, LiNi_0.93_Ti_0.07_O_2_ exhibits significantly improved long‐term cycling stability compared to pristine LiNiO_2_.

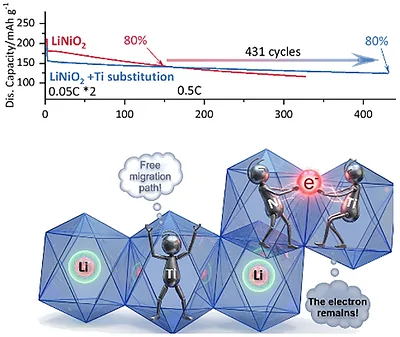

## 1 Introduction

Lithium‐ion batteries (LIBs) are adopted as the primary energy storage system in portable electronics and electric vehicles (EVs), supporting the global transition away from internal combustion engines and helping to mitigate CO_2_ emissions [1, 2, 3]. A key factor limiting their performance, however, is the performance and costs of cathode materials [4, 5, 6, 7, 8, 9]. In recent years, high‐Ni (LiNi_x_ *TM*_1‐x_O_2_, *x* ≥ 0.8, where *TM* is transition metal) layered oxide materials such as lithium nickel cobalt aluminum oxide (LiNi_x_Co_y_Al_z_O_2_, NCA) and lithium nickel manganese cobalt oxide (LiNi_x_Mn_y_Co_z_O_2_, NMC) have gained considerable attention, due to their high capacity and reduced cost compared to lithium cobalt oxide (LiCoO_2_, LCO) [10, 11]. However, despite their advantages, most high‐Ni materials still rely on Co, a costly and scarce metal with limited global reserves. The uneven distribution of Co across different geographical regions and the associated risks in raw material procurement further complicate its widespread use [12, 13].

These challenges underscore the growing need for Co‐free alternatives that can deliver high performance while ensuring sustainability and economic viability. However, in high‐Ni layered oxide cathodes, cobalt plays a crucial role in stabilizing the structure. It is commonly incorporated to suppress Ni/Li cation mixing, primarily due to its “magnetic frustration” property, which can reduce the likelihood of Li^+^ and Ni^2^^+^ exchanging positions, thereby preserving the layered structure and enhancing long‐term electrochemical stability [14]. As a result, even though Co‐free high‐Ni layered oxides have attracted great attention, they often face inherent structural degradation. These issues include not only Ni/Li cation disorder, but also the formation of residual lithium compounds and irreversible phase transitions (Ni─O‐like rock‐salt structure formation on the surface), all of which negatively impact cycling performance [12, 15].

In this work, Ti is investigated as a Co‐free stabilizing element in high‐Ni layered oxides, owing to its abundance, low cost, stable tetravalent state, and strong Ti─O bonds. To date, only a limited number of studies have examined LiNi_1‐x_Ti_x_O_2_ (LNTO, 0 < *x* < 0.2) [16, 17]. These reports indicate that Ti substitution enhances the structural integrity and cycling stability of LiNiO_2_, although excessive Ti content reduces the specific capacity due to the electrochemical inactivity of Ti^4+^. LiNiO_2_ is intrinsically prone to cooperative Jahn–Teller distortion, which is closely linked to phase transitions, lattice distortion, and cycling‐induced degradation [18, 19]. More recent studies on Ti‐containing Co‐free LiNiO_2_‐based cathodes and related dopant‐stabilized layered oxides further suggest that structural stabilization can be achieved through dopant distribution control [20], dopant‐induced cation/defect ordering [21], microstructural engineering [22, 23], suppressed phase transitions [24], and pillar‐type framework stabilization [25, 26].

Despite these advances, the atomic‐scale origin of Ti‐induced stabilization remains insufficiently resolved. Most previous studies have correlated Ti substitution with improved electrochemical performance, suppressed structural degradation, or surface stabilization, but the fundamental electronic and local structural roles of Ti within the Ni─O framework remain unclear. In particular, how Ti affects the Ni─O electronic structure and the cooperative Jahn–Teller distortion of NiO_6_ octahedra has not been directly established.

Here, we disentangle the electronic and structural roles of Ti within the Ni─O framework. Specifically, we investigate (i) the electron‐withdrawing effect of Ti on the Ni─O local electronic structure, and (ii) its influence on the cooperative Jahn–Teller distortion across NiO_6_ octahedra, which governs phase stability and degradation in LiNiO_2_‐derived cathodes. Near‐edge X‐ray absorption fine structure (NEXAFS) spectroscopy, combined with density functional theory (DFT)‐based band structure calculations, is used to resolve the electronic effects of Ti incorporation. In parallel, extended X‐ray absorption fine structure (EXAFS), high‐resolution transmission electron microscopy (HRTEM), and powder diffraction (PD) are employed to correlate local electronic changes with octahedral ordering across multiple length scales. Together, these results provide an atomic‐scale mechanistic description of Ti‐induced stabilization of the crystallographic and electronic structure, moving beyond empirical correlations between dopant substitution and cycling performance.

## 2 Results and Discussion

### 2.1 Structural and Electrochemical Characterizations

LNO (LiNiO_2_), LNTO‐1 (LiNi_0.93_Ti_0.07_O_2_), and LNTO‐2 (LiNi_0.86_Ti_0.14_O_2_) were synthesized using the conventional co‐precipitation method, which offers the potential for scale‐up for industrial applications. The total Ti content in the synthesized materials is determined by inductively coupled plasma optical emission spectroscopy (ICP‐OES, Table S1). The particle size analysis (PSA) results in Figure S1 reveal that the mean secondary particle size distributions across all these materials are roughly comparable. As shown in Figure 1a, all synthesized materials are phase‐pure as confirmed by powder diffraction. The diffraction peaks are well‐defined, indexable to a NaFeO_2_‐type rhombohedral structure, corresponding to the $R\bar{3}m$ space group. As Ti incorporation increases, a systematic leftward shift is observed for all reflections, such as 003 and 018/110 doublets, indicating successful Ti substitution into the rhombohedral lattice. Further structural analysis using Rietveld refinement (see Figure S2; refinement sequences can be found in Supporting Information) was carried out. As reported in the literature, Ti can occupy both transition‐metal (*TM*) and Li lattice sites [27]. Accordingly, three different models were used for the refinement: (a) Ti distributed over both the *TM* and Li sites, (b) Ti occupying only the *TM* site, and (c) Ti occupying only the Li site. The refinement results shown in Figure S3 indicate that model (a) provides the best match among the three models. These results suggest that, in the Ti‐substituted samples, Ti is distributed over both the *TM* and Li sites. Partial Ti occupation at Li sites may contribute to interslab stabilization through a pillar‐like effect. The Li/Ni disorder values are estimated to be 1.4% for LNTO‐1 and 2.2% for LNTO‐2, compared to 1.7% for unsubstituted LNO (see Tables S2–S5).

**FIGURE 1:**
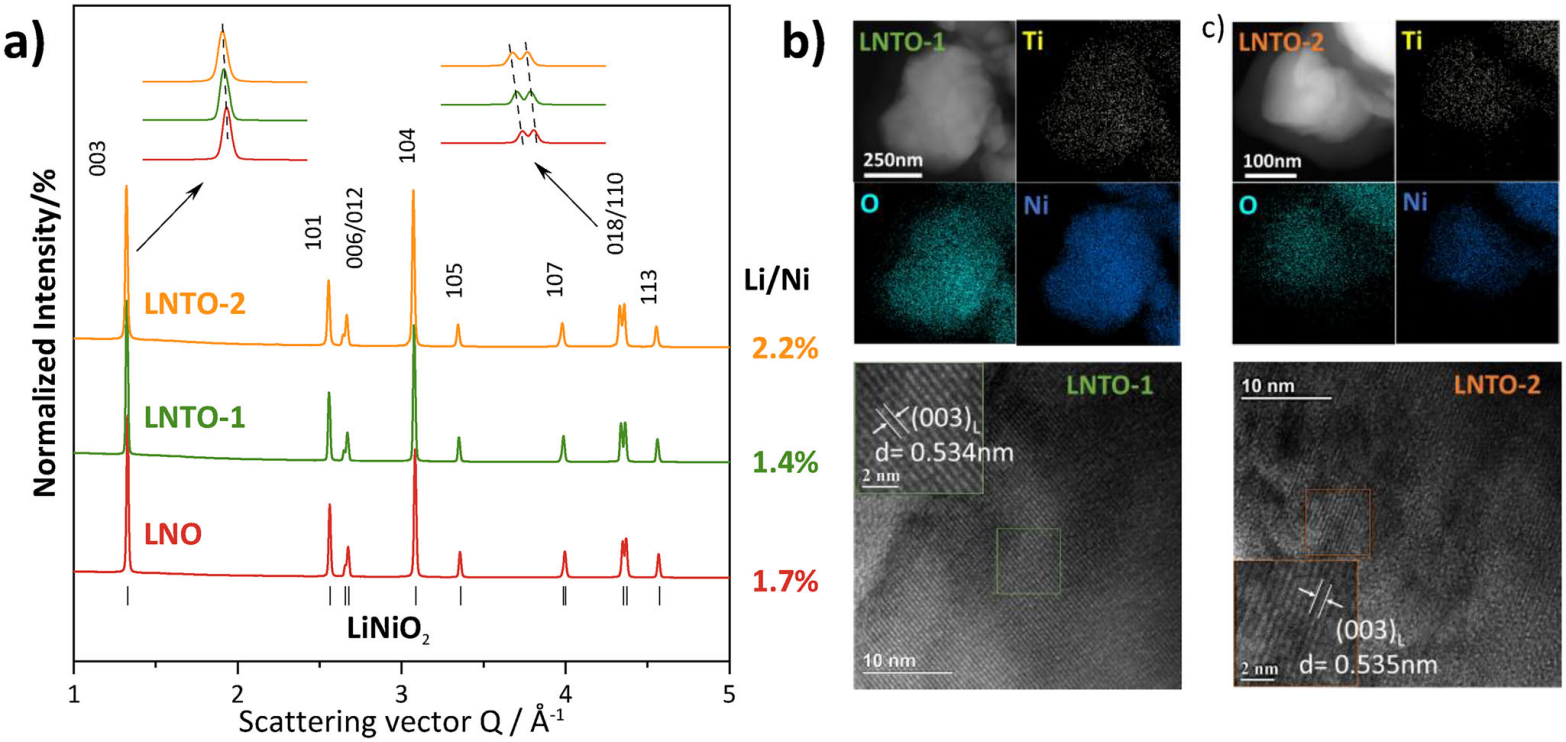
Rhombohedral structure of Ti‐substituted LiNiO_2_ (LNTO‐1 and LNTO‐2), same as LNO. (a) Synchrotron X‐ray powder diffraction (SXPD) patterns of LNO, LNTO‐1, and LNTO‐2. (b) Elemental mapping and HRTEM images of LNTO‐1, with lattice spacings determined by fast Fourier transform (FFT) analysis of the marked regions. (c) Elemental mapping and HRTEM images of LNTO‐2.

The structural origin of Ti‐induced stabilization is further discussed in the following sections based on combined operando SXPD, EXAFS, and HRTEM analyses. With increasing Ti content, the *a* (= *b*), and *c* lattice parameters show a corresponding increase. Elemental mapping using scanning electron microscopy coupled with energy‐dispersive X‐ray spectroscopy (SEM‐EDX) (Figure S4) confirms a homogeneous distribution of Ti across the secondary particles. Additionally, high‐resolution transmission electron microscopy images (HRTEM, Figure 1b,c; Figure S5) demonstrate that Ti is uniformly distributed not only within the secondary particles but also throughout the primary particles of both LNTO‐1 and LNTO‐2. Moreover, these HRTEM maps reveal that LNTO‐1, LNTO‐2, and LNO all retain a well‐ordered layered structure. The calculated lattice distances align well with the expected lattice *d*‐spacing of the (003) planes in rhombohedral structures [28, 29, 30], denoted as (003)_L_ in the insets. The d‐spacing of the (003) planes increases from 0.473 nm in LNO to 0.534 nm in LNTO‐1, and further slightly increases to 0.535 nm in LNTO‐2. This trend indicates that Ti substitution enlarges the Li slab spacing within the layered structure, which is consistent with the left shift of the (003) reflections observed with PD, Figure 1a.

The electrochemical performance of LNO, LNTO‐1, and LNTO‐2 is presented in Figure 2. The materials are cycled vs. Li/Li^+^ within a voltage window of 3.0–4.4 V. As shown in Figure 2a (rate performance), LNO delivers the highest initial specific discharge capacity of 210 mAh g^−^^1^ at 0.05C, followed by LNTO‐1 (192.8 mAh g^−^^1^) and LNTO‐2 (138.7 mAh g^−^^1^). However, both LNTO‐1 and LNTO‐2 outperform LNO in rate capability. After 45 cycles in the rate test, LNTO‐1 and LNTO‐2 recover to 194.4 and 140 mAh g^−^^1^, respectively, values that even slightly exceed their initial capacities, while LNO could only recover to 189.6 mAh g^−1^, noticeably lower than its initial 210 mAh g^−^^1^.

**FIGURE 2:**
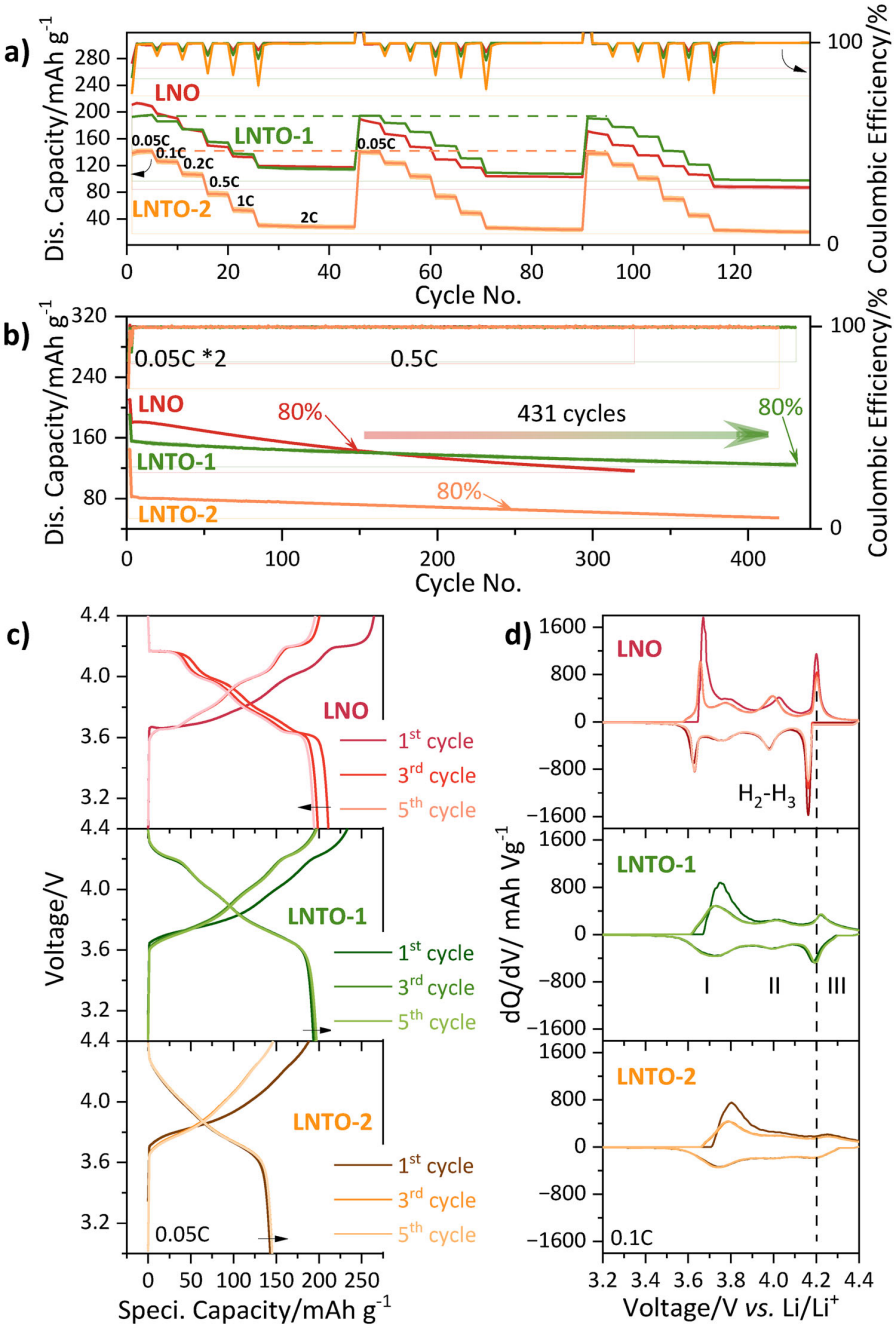
Battery performance of Ti‐substituted LiNiO_2_ (LNTO‐1 and LNTO‐2) compared with LNO. (a) rate performance and (b) cycling stability in the voltage window of 3.0–4.4 V. The first two cycles were conducted at a constant current of 9 mA g^−1^ (0.05C rate), followed by cycling at 90 mA g^−1^ (0.5C rate). Both (a) and (b) include error bars. (c) Voltage versus specific capacity and (d) dQ/dV vs. voltage curves for the first, third, and fifth cycles. 1C corresponds to 180 mAh g^−1^.

In the cycling stability test shown in Figure 2b, LNTO‐1 exhibits the most robust performance, retaining 80% of its capacity after 431 cycles at a 0.5 C rate. In contrast, LNO and LNTO‐2 reach the same retention after only 146 and 247 cycles, respectively, highlighting the superior long‐term stability of LNTO‐1. As shown in Figure 2c, even within the first 5 cycles at 0.05 C, LNO exhibits a noticeable specific capacity loss of 16.7 mAh g^−^^1^, decreasing from 211 to 194.3 mAh g^−^^1^. In contrast, the LNTO samples show a slight capacity recovery: LNTO‐1 rises by 3.9 mAh g^−^^1^ (from 193.6 to 197.5 mAh g^−^^1^) and LNTO‐2 increases by 2.2 mAh g^−^^1^ (143–145.2 mAh g^−^^1^), demonstrating superior initial capacity retention. The calculated initial Coulombic efficiencies (ICE) are 79.8% for LNO, 83.0% for LNTO‐1, and 76.0% for LNTO‐2, respectively, with LNTO‐1 exhibiting the highest value. Since a higher ICE has been reported to correlate with improved cycling stability [31], this result further supports the superior electrochemical performance of LNTO‐1.

The reduced specific capacity upon Ti substitution is further reflected in Figure 2d. The integrated areas of the reaction peaks, which correspond to the specific capacities in Figure 2c, decrease progressively from LNO to LNTO‐1 and LNTO‐2. This trend indicates that increasing Ti content reduces the number of redox‐active species owing to the electrochemical inactivity of Ti^4+^. Therefore, Ti substitution introduces a trade‐off between structural stabilization and specific capacity. While a moderate Ti content enhances cycling stability, excessive Ti substitution does not provide further benefit. Partial Ti occupation at Li sites may be considered a relatively stable form of local cation disorder that contributes to framework stabilization; however, excessive Ti incorporation further increases the degree of cation disorder, which is generally unfavorable for efficient Li^+^ transport and electrochemical reversibility and may ultimately lead to a phase transformation from the rhombohedral structure toward a more cubic‐like structure [32]. To further investigate the optimal Ti substitution level, additional compositions were synthesized and evaluated, with the corresponding results presented in Figures S6 and S7. Based on these results, x ≈ 0.07 provides the best balance between structural stabilization, cycling stability, and electrochemical activity in the present system.

### 2.2 Suppression of Phase Transition

The voltage profile in Figure 2c reveals that LNO exhibits three distinct voltage plateaus at 3.63, 4.0, and 4.17 V. During lithium extraction, LNO undergoes a sequence of reversible and stable phase transitions: H_1_ → M → H_2_ → H_3_ [33, 34, 35], as shown in Figure 3a. Herein, H_1_ represents the initially hexagonal phase, M corresponds to a monoclinic phase, H_2_ is a second hexagonal phase, and H_3_ is a third hexagonal phase. According to the Gibbs phase rule [36], the chemical potential of the LNO cathode material remains almost unchanged during phase transitions, resulting in three voltage plateaus observed in Figure 2c. Each phase transition region corresponds to a plateau, which in turn leads to distinct reaction peaks in the dQ/dV curve (Figure 2d), specifically associated with the H_1_‐M, M‐H_2_, and H_2_‐H_3_ phase transitions. These are all classified as first‐order phase transitions, as they involve phase coexistence, a key characteristic of first‐order behavior [37]. Typically, such transitions are accompanied by latent heat and require overcoming a significant energy barrier between phases. In this context, the transitions involve collapse of interlayer repulsion and slab gliding in Li‐depleted regions, which are consistent with the abrupt structural changes expected in first‐order transitions.

**FIGURE 3:**
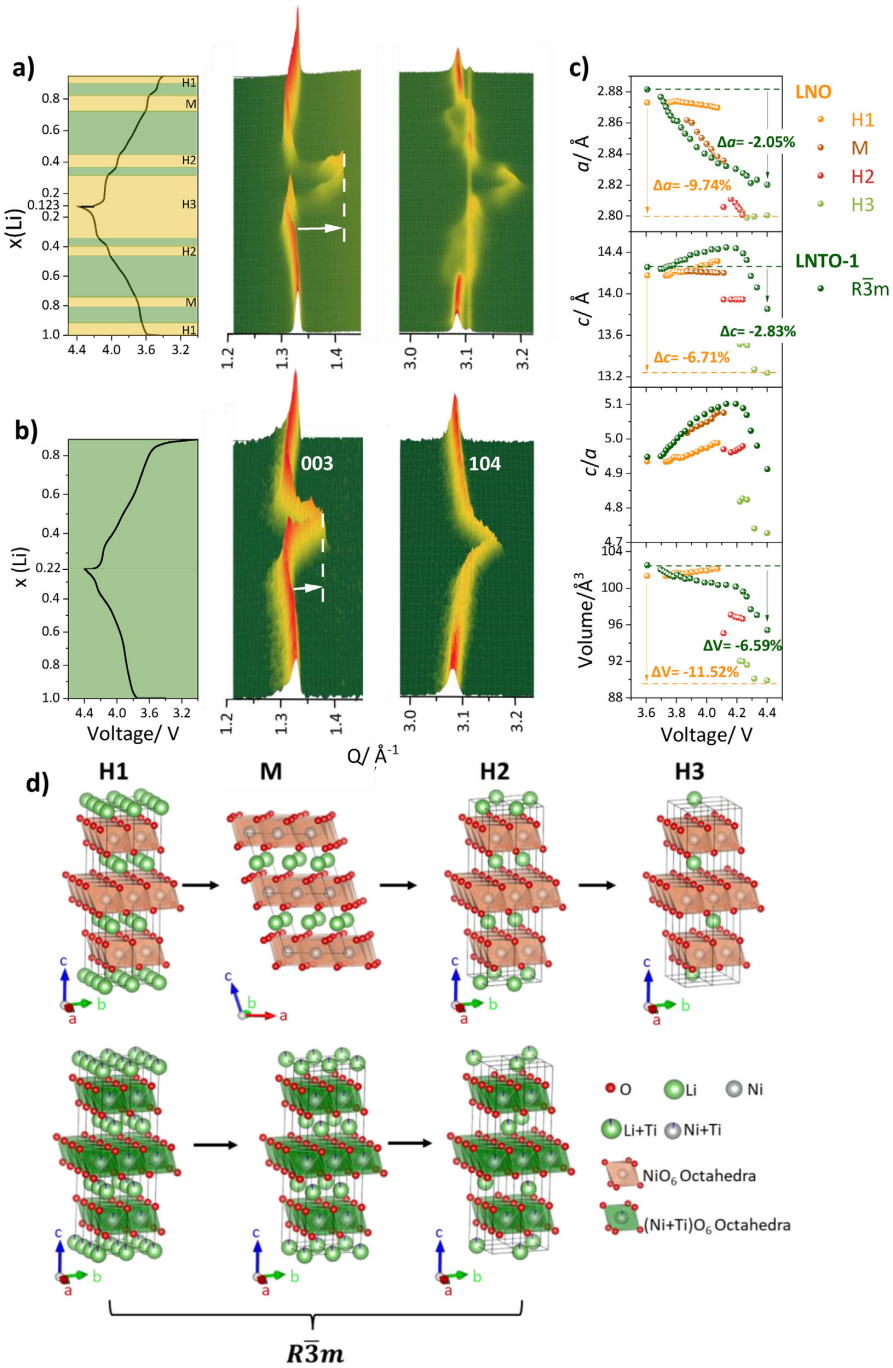
Lattice structure analysis *via operando* SXPD reveals that Ti substitution alleviates the lattice strain induced by phase transitions. (a) 3D contour plots of *operando* SXPD patterns for LNO. The phases H1, M, H2, and H3 are marked. The transition zone between each stable phase region serves as a phase transformation region, encompassing not only the preceding phase but also the new phase. (b) 3D contour plots of *operando* SXPD patterns for LNTO‐1. Since no phase transformation is observed, only a single‐phase $R\bar{3}m$ is present, with reflections corresponding to this phase—such as 003, 104—marked in the diagram. (c) Shrinkage of *a* = *b*, *c* lattice parameters, *c*/*a*, and unit cell volume during charging with standard deviations. The evolution of the unit cell volume for the M phase has not been included. More detailed SXPD patterns can be found in Figures S9–S11. (d) Schematic representation of the structural evolution of LNO and LNTO‐91 during the delithiation process.

In contrast, LNTO‐1 and LNTO‐2 display no clear voltage plateaus in Figure 2c. The absence of voltage plateaus in the voltage profile of LNTO‐1 aligns with the *operando* SXPD measurements in Figure 3b, which show no distinct phase transformation. Instead, a continuous shift of the rhombohedral reflections (space group $R\bar{3}m$) is observed, including the 003, 104 reflections. Consequently, the reaction peaks in Figure 2d are more appropriately labeled as reaction peaks I, II, and III, rather than using nomenclature based on phase transformations.

Rietveld refinement is carried out for these *in‐situ* SXPD patterns, and the evolution of the *a*, *c* lattice parameters, *c/a* ratio, and unit cell volume is summarized in Figure 3c. In the absence of structural distortion, the lattice parameters of the monoclinic (M) and hexagonal (H) phases are related by the following expressions: *a_M_* =  √3*a_H_*, *b_M_* =  *a_H_*, *c_M_* =  *c_H_*/3*sin*β, and β  =  180 − *tan* ^−1^ *c_H_*/√3*a_H_* [38]. Under these conditions, the M and H lattice parameters can be directly compared within the same diagram, except for the unit cell volumes (Figure 3c). Upon delithiation, LNO shows discontinuous contraction in *a* lattice parameter, i.e., shrinking by 9.74% from 2.87551(7) Å to 2.80044(10) Å. In contrast, LNTO‐1 exhibits a continuous change, with the *a* parameter decreasing by 2.05% from 2.87927(5) Å to 2.82013(4) Å. Similarly, the *c* lattice parameter of LNO changes non‐monotonically, first increasing and then decreasing, resulting in a total contraction of 6.71% (from 14.19020(50) Å at 0%SOC to 13.23774(63) at 100%SOC Å). In comparison, LNTO‐1 displays a smoother and more gradual *c*‐axis contraction, with a total change of 2.83% (from 14.22812(33) Å at 0%SOC to 13.85416(354) Å at 100%SOC). The shrinkage of unit cell volume is a critical indicator of overall crystal contraction and is closely linked to the risk of particle cracking during cycling [39]. For LNO, the volume change is again discontinuous, dropping from 101.613(1) Å^3^ to 89.908(6) Å^3^, corresponding to ΔV = 11.52%. In contrast, LNTO‐1 shows a more gradual volume reduction, from 102.151(1) Å^3^ to 95.422(31) Å^3^, with ΔV = 6.59%—nearly half the volume change observed in LNO.

The *c/a* ratio serves as an important indicator of shear strain within the lattice, which can lead to anisotropic stress, ultimately causing structural collapse and particle cracking [27, 40]. In LNO, the *c/a* ratio changes abruptly, particularly across the H2–H3 transition, where it drops sharply from 4.98 to 4.82, and then continues to decrease to 4.73. This behavior reflects a nonlinear and discontinuous lattice response. In contrast, LNTO‐1 shows a smooth and continuous change in the *c/a* ratio, without any sudden jumps. An abrupt contraction observed in LNO generates mechanical shear stress within the layered structure, significantly increasing the likelihood of crack formation and structural degradation. Notably, this sharp collapse is most pronounced during the H2–H3 phase transition, aligning with literature [33]. Thus, the H2–H3 transition is highly detrimental upon long‐term cycling.

*Operando* SXPD shows that LNTO‐1 does not undergo distinct phase transitions, in contrast to the clear, multi‐phase transitions observed in LNO. This indicates that Ti substitution effectively suppresses phase transitions, particularly the H2–H3 transition, and it alters the reaction pathway toward a more continuous (or single‐phase) process. The partial substitution of Ni by Ti also reduces mechanical stress within the lattice upon cycling, thereby minimizing structural degradation. As a result, this structural stabilization contributes directly to the enhanced cycling stability observed in LNTO‐1.

The electrode morphologies of LNO and LNTO‐1 after 150 cycles are investigated by SEM. As shown in Figure S8, clear particle cracks are observed in LNO, whereas such cracks are largely absent in LNTO‐1. This is a macroscopic manifestation of mechanical degradation that originates from accumulated lattice strain and volume changes during cycling [41]. These findings are consistent with the long‐term cycling performance shown in Figure 2b, where LNTO‐1 outperforms LNO. To elucidate the atomistic origin of the enhanced structural stability upon Ti incorporation, both the crystallographic and electronic structures are investigated in more detail.

### 2.3 Cooperative Suppression of Jahn–Teller Distortion

To further investigate how Ti alters the crystallographic structure and potentially suppresses the phase transitions, *operando* hard X‐ray absorption spectroscopy (XAS) [42] is conducted on LNO and LNTO‐1 during the first cycle to unravel the local ordering around the transition metals (overview Figure S12). Figure 4a–c presents the Fourier transform (FT) magnitudes of k^3^‐weighted hard XAS spectra. The samples at OCV, charged to 4.4 V, and discharged to 3.0 V are selected for data fitting with the Artemis software [43], Figure 4d–f (see Tables S7–S9 for details). The first shell in the Ni K edge (Figure 4a,b) and Ti K edge (Figure 4c) corresponds to the nearest O atoms, with the distance reflecting the Ni─O or Ti─O bond length, while the second shell relates to the nearest *TM* atoms, with the distance reflecting the Ni─*TM* or Ti─*TM* bond length. As indicated by the arrow in Figure 4a,b, the Ni─O bond length slightly decreases while the corresponding EXAFS intensities increase during delithiation in both LNO and LNTO‐1. This behavior is attributed to the oxidation of Ni, which results in a reduction of its ionic radius from 0.69 Å (Ni^2+^) to 0.62 Å (Ni^4+^). As the Ni─O bond shortens, the intensity of the Fourier Transform of the EXAFS signal increases, since the amplitude is approximately inversely proportional to the square of the bond length (∝ 1/R^2^), assuming other factors are constant. In contrast, the Ti─O bond exhibits a decrease in EXAFS intensity during delithiation (Figure 4c), indicative of a slight elongation of the Ti─O bond. This opposing trend indicates a compensatory structural response of TiO_6_ octahedra to the contraction of NiO_6_ units.

**FIGURE 4:**
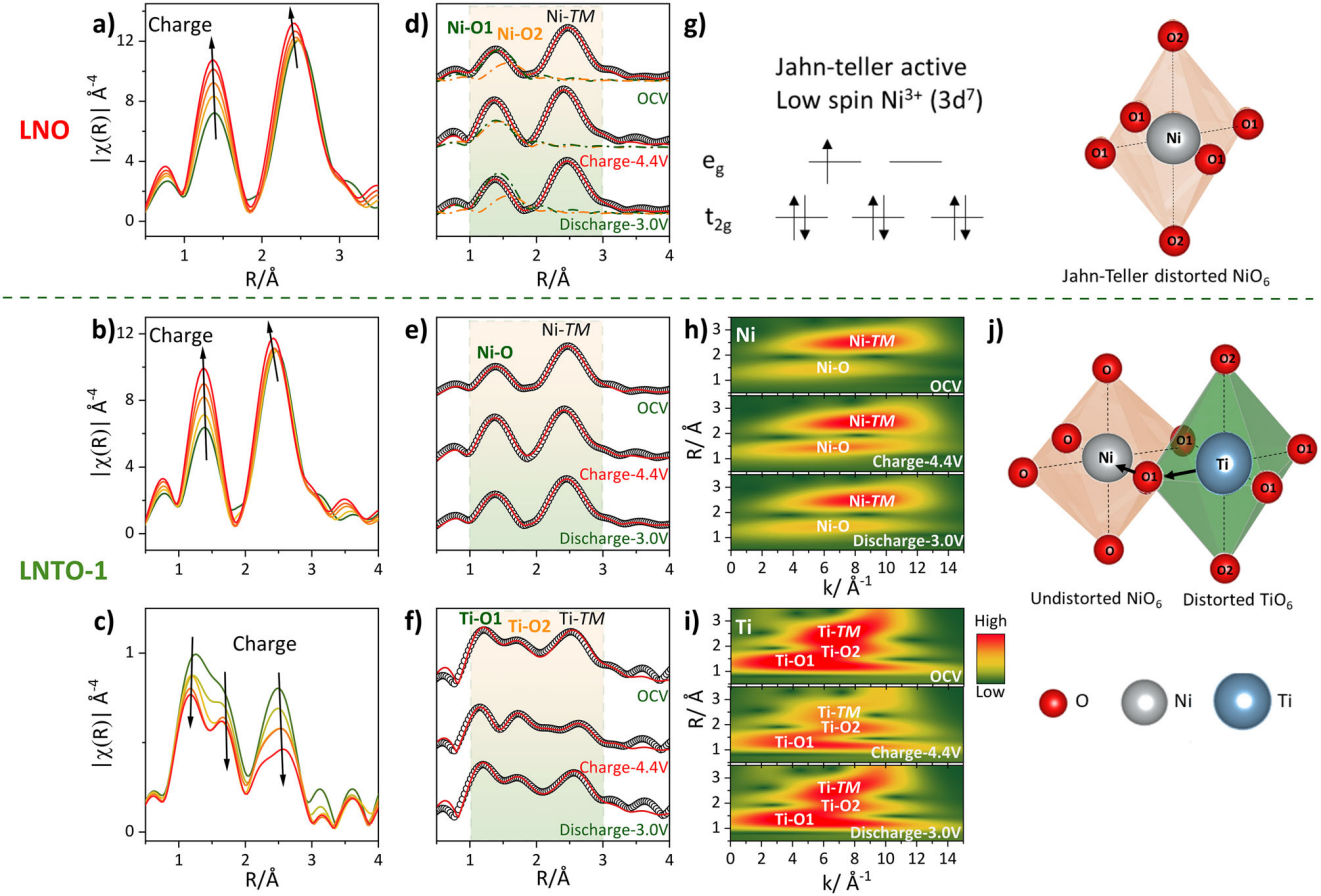
*Operando* extended X‐ray absorption fine structure (EXAFS) technique reveals that no obvious Jahn–Teller distortion is observed in Ti‐substituted LNO. Fourier‐transformed (FT) magnitudes of k^3^‐weighted EXAFS spectra at the Ni K‐edge for (a) pristine LNO and (b) Ti‐substituted LNTO‐1, and at the Ti K‐edge for (c) LNTO‐1, recorded during the charging process in the first cycle. EXAFS fitting profiles at the Ni K‐edge for (d) LNO and (e) LNTO‐1, and at the Ti K‐edge for (f) LNTO‐1, collected at open‐circuit voltage (OCV), after charging to 4.4 V, and after discharging to 3.0 V in the first cycle. Light yellow area indicates the fitting regions. (g) Schematic illustration of Jahn–Teller distorted NiO_6_ octahedra. Wavelet‐transformed EXAFS diagrams of LNTO‐1 at the (h) Ni K‐edge and (i) Ti K‐edge. (j) Schematic showing how TiO_6_ octahedra alleviate Jahn–Teller distortion in neighboring NiO_6_ units. See also Figures S12–S15 and Tables S7–S9 for additional details.

The presence of low‐spin Ni^3+^ (3d^7^: t_2g_^6^e_g_^1^, Figure 4g) in LNO induces Jahn–Teller distortions [44, 45], which destabilize the lattice, trigger phase transitions, and cause abrupt volume changes [46], as shown in Figure 3. These structural instabilities can lead to microcrack formation, surface pulverization, and ultimately result in capacity loss [45]. Low‐spin Ni^3+^ in octahedral coordination is susceptible to spontaneous symmetry breaking to lower the system's energy, through tetragonal elongation or compression (Figure 4g). This distortion splits the first Ni─O shell into two distinct shells: Ni─O_1_ and Ni─O_2_ [44, 46], as illustrated in Figure 4d. To verify this effect, Figure S13 presents EXAFS fitting results using two models: one with two single scattering paths (Ni─O and Ni─*TM*) and another with three single scattering paths (Ni─O_1_, Ni─O_2_, and Ni─*TM*). The two‐paths model fails to adequately capture certain regions of the data compared to the three‐paths model, resulting in a higher R‐factor relative to the three‐paths model. This supports the presence of Jahn–Teller distortion in LNO. With the Ti substitution, Figure S14 shows the same two fitting models applied to LNTO‐1. However, in this case, the three‐paths model does not improve the fitting and fails to capture key regions compared to the two‐paths model, yielding a higher R‐factor. This outcome confirms the absence of Jahn–Teller distortion in LNTO‐1 (Figure 4e). In Figure 4f, the Ti K‐edge fit clearly displays three distinct coordination shells within the 1–3 Å region. Accordingly, a three‐path model is used for Ti EXAFS fitting, and the results are in excellent agreement with the experimental data.

Wavelet‐transformed EXAFS enhances visualization by combining spatial (R‐space) and frequency (k‐space) information Figure 4h. The Ni K‐edge of LNTO‐1 displays two distinct intensity maxima, corresponding to two coordination shells. In contrast, the Ti K‐edge in Figure 4i clearly exhibits three distinct maxima, which are labelled in the diagram. However, it is important to note that the number of intensity maxima in a wavelet transform does not necessarily correspond to the number of coordination shells, as contributions from different scattering paths can overlap in both R‐ and k‐space. For example, in Figure S15, LNO also shows two distinct signals, but the first signal arises from the combined contribution of a Ni‐O_1_ and Ni‐O_2_ shell.

In Figure 4a,b and Figure S15, the Ni K‐edge of both LNO and LNTO‐1 becomes stronger upon charge from OCV to 4.4 V and then returns to their original states upon discharge to 3.0 V, demonstrating a reversible process. Figure 4c shows that the Ti K‐edge signals of LNTO‐1 behave oppositely during cycling but still exhibit reversibility. This contrasting behavior supports the idea that Ti plays a compensatory structural role in response to changes occurring in the Ni environment.

As previously mentioned, Ti can occupy both the 3a site (*TM* site) and the 3b site (Li site) within the crystal structure (see Figure S16). When Ti occupies Li sites, the local TiO_6_ units remain structurally connected to the surrounding NiO_6_ framework through shared oxygen atoms across adjacent slabs, which may impose a local structural constraint on the evolution of cooperative Jahn–Teller distortion. Therefore, regardless of the occupied site, TiO_6_ can still interact with the surrounding NiO_6_ framework and inhibit Jahn–Teller distortions of NiO_6_ through local structural compensation and enhanced lattice rigidity. Based on this mechanism, a schematic illustration depicting the structural interaction between NiO_6_ and TiO_6_ is presented in Figure 4j.

In conclusion, while LNO undergoes a cooperative Jahn–Teller distortion that ultimately leads to phase transformations during cycling, Ti incorporation into the Ni─O host structure suppresses these distortions, resulting in single‐phase Li‐ion intercalation/deintercalation.

### 2.4 Electronic Structure Stabilization

The effect of Ti substitution on the electronic structure is investigated with NEXAFS and band structure calculations using DFT. The Ni K‐ and Ti K‐edge of the pristine samples as well as upon charge and discharge are presented in Figure S12. The Ni spectra display a broad absorption maximum at ∼8350.6 eV and a pre‐edge feature at ∼8330 eV for all samples (LNO, LNTO‐1, and LNTO‐2). The main edge corresponds to the dipole‐allowed 1s–4p transition, while the pre‐edge is associated with a formally forbidden 1s–3d or 4p transition, commonly observed in distorted octahedral Ni sites [47, 48, 49]. Figure S12a shows that the overall spectral shapes of the pristine samples remain largely unchanged with Ti incorporation. However, the energy shifts toward higher values indicate an oxidation of the *TM*─O host structure [50, 51, 52]. Moreover, the shift of the main edge toward lower energies with increasing Ti content suggests that Ti has an electron‐withdrawing effect on the Ni‐O lattice. Figure S12b,c demonstrates that the spectral shapes of both LNTO‐1 and LNO remain largely unchanged upon cycling, whereas the peak energy changes. The Ni K‐edge in LNTO‐1 shifts from 8347.87 to 8350.34 eV upon charge and returns to 8347.90 eV after the first cycle, while LNO shows a comparable reversible shift from 8347.81 to 8347.90 eV (Table S6). These results indicate a nearly reversible Ni redox process in both materials during the initial cycle, with LNTO‐1 exhibiting slightly improved reversibility. This trend is consistent with the electrochemical cycling data in Figure 2.

Similar to the Ni K edge, the Ti K edge exhibits a pre‐edge absorption feature, Figure S12d, which is typically associated with distorted octahedral Ti [53]. For reference TiO_2_ (anatase), in the first pre‐edge peak (A_1_) is primarily attributed to quadrupolar transitions to the *t*_2g_ levels of the Ti octahedron, while the second (A_2_) and third (A_3_) pre‐edge features correspond to 1s → 3d dipolar transitions [54]. Additionally, the shoulder B_1_ is assigned to the 1s to 4p transition [55]. The pre‐edge peak of LNTO‐1 and LNTO‐2 reveals two components located at 4968.4 eV (A_1_) and 4970.7 eV (A_2_), indicating that the spectral shape is influenced by the octahedral coordination environment. The sharp main absorption feature at ∼4987 eV is attributed to the dipole‐allowed 1s → 3p transition. As shown in Figure S12e, the overall spectral shapes exhibit slight variations during cycling. A small shoulder peak at ∼4982.5 eV appears at high SOCs but is absent at low SOCs. Additionally, the pre‐edge shape changes slightly with increasing SOCs, suggesting that the Ti environment becomes distorted upon charge. This distortion may compensate for microstrain that typically develops at high SOCs when the lattice begins to break down, Figure 4c (at >4.2 V). The evolution of the Ti K pre‐ and main‐edge features does not follow a consistent trend, which may also be attributed to this structural distortion, Figure S12 and Table S6.

To further investigate the effect of Ti substitution on the electronic structure of LNO, the band structure is investigated with DFT calculations. As shown in Figure 5, the PDOS of pristine LNO reveals strong hybridization between Ni 3d and O 2p states near the Fermi level. Upon Ti substitution, in LNTO‐1, additional Ti 3d states appear in the conduction band region, while the overall distribution of Ni 3d and O 2p states is slightly modified (Figure 5b). To quantitatively evaluate the electronic structure, the band centers of Ni 3d and O 2p states were extracted from the PDOS (Figure 5c). For pristine LNO, the energy separation between Ni 3d and O 2p band centers (Δε_d–p_) is calculated to be 0.517 eV. After Ti substitution, this value increases to 0.697 eV, indicating an enlarged Ni 3d‐O 2p energy separation. Such an increase in Δε_d–p_ suggests a modulation of the Ni─O electronic interaction and a slight reduction in the Ni─O covalency and thus confirms the electron‐withdrawing effect of Ti substitution. The modified electronic structure can influence the local structural stability of the layered lattice. Weakened Ni─O hybridization can reduce the Jahn–Teller driving force, thereby stabilizing the NiO_6_ octahedra. Combined with the experimental observations from EXAFS in Figure 4, which reveal a compensatory evolution of Ti─O and Ni─O bond changes during cycling, these results suggest that TiO_6_ octahedra act as local structural stabilizers that accommodate lattice strain and mitigate the distortion of neighboring NiO_6_ units.

**FIGURE 5:**
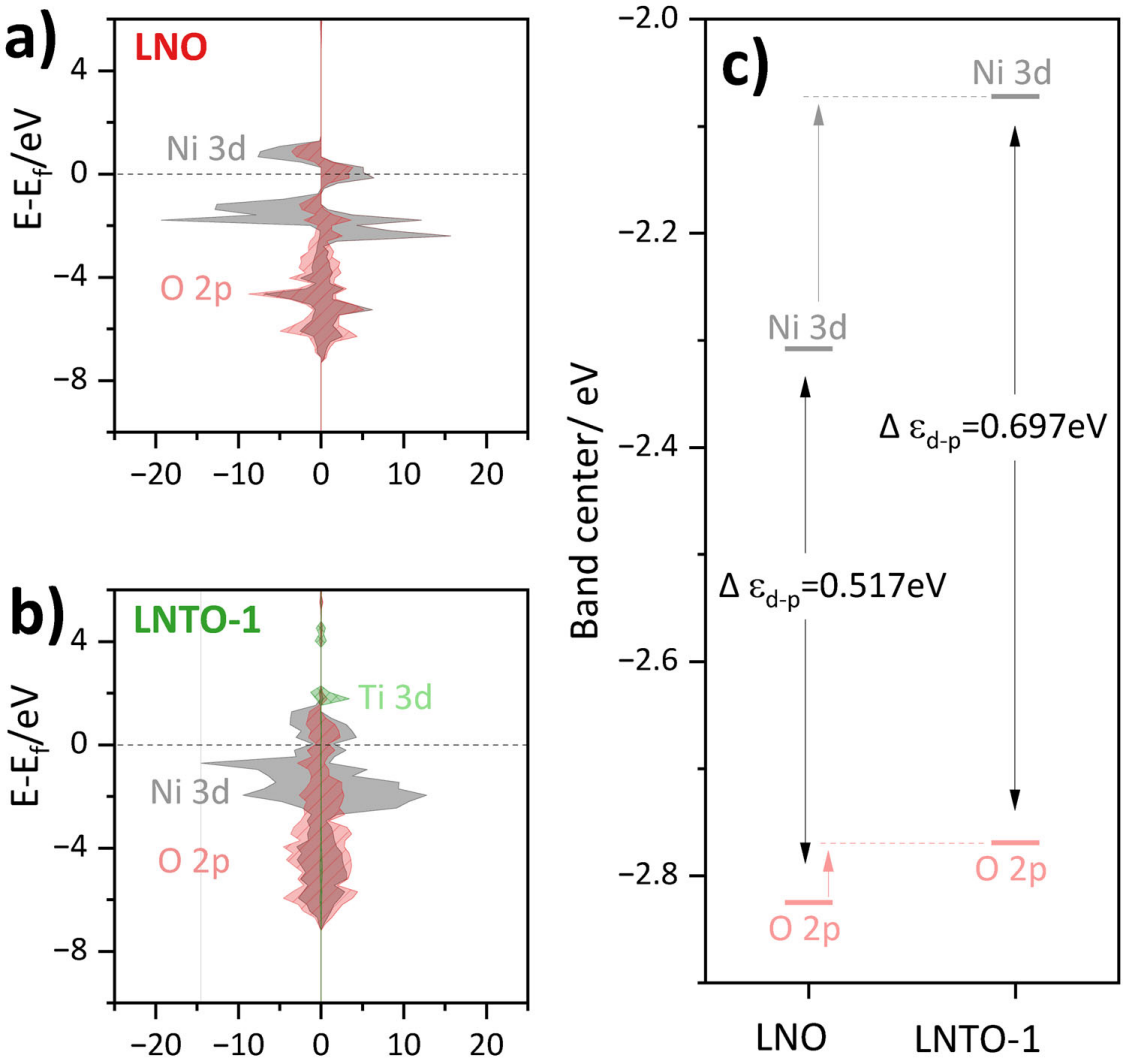
Electronic structure evolution revealed by Ni 3d and O 2p band centers upon Ti substitution. Density functional theory (DFT)‐calculated projected density of states (PDOS) of (a) LNO and (b) LNTO‐1. (c) Calculated band centers of Ni 3d and O 2p states and the corresponding Ni‐3d─O‐2p energy separation (Δε_d‐p_). The enlarged d–p separation suggests a modified Ni─O electronic interaction, which is expected to influence the local structural stability of the layered lattice.

To further verify the electron‐withdrawing effect induced by Ti incorporation in LNO, *TM* L‐edge and O K‐edge spectra (Figure 6; Figure S17) were collected to probe the occupied redox‐active valence states of the material. Employing NEXAFS at the *TM* L and O K edges provides valuable information about the oxidation states of 3d metals and O 2p states as well as about the electronic nature of the *TM*─O bonds [48, 56, 57, 58, 59]. The NEXAFS measurements were performed in fluorescence yield (FY) mode with a sampling depth of ∼300 nm on LNO and LNTO‐1 at different states of charge (SOCs) during the first cycle applying a C/20 rate [48]. Specific SOCs were selected during discharge, including 0%, 50%, 75%, 80%, and 100%. Notably, 80% SOC was chosen as it corresponds to the maximum reaction peak III in the dQ/dV versus voltage plot (Figure 2). The pre‐edge peaks O_1_ (∼529 eV) and O_2_ (∼530 V) in the O K edge (Figure 6a,b) are present because of electron holes in the O 2p band formed due to a hybridization of *TM t*_2g_ or *e*_g_ with O 2p orbitals [56]. The comparison of the O K‐edge spectra for pristine LNO and LNTO‐1 is shown in Figure S18a. The enhanced O K‐edge pre‐edge intensity of LNTO‐1 cannot be simply attributed to a higher Ni oxidation state. Instead, it is more reasonably associated with the introduction of Ti^4+^ (3d^0^), which provides additional unoccupied Ti 3d states hybridized with O 2p orbitals. Together with the redistribution of hybridized O 2p–*TM* 3d unoccupied states revealed by the DFT calculations (Figure 4), these Ti‐related electronic states likely contribute to the enhanced pre‐edge feature observed in LNTO‐1. Similar modulation of the oxygen electronic structure through strong heteroatom–oxygen interactions has also been reported in other doped high‐Ni layered oxide systems [60, 61].

**FIGURE 6:**
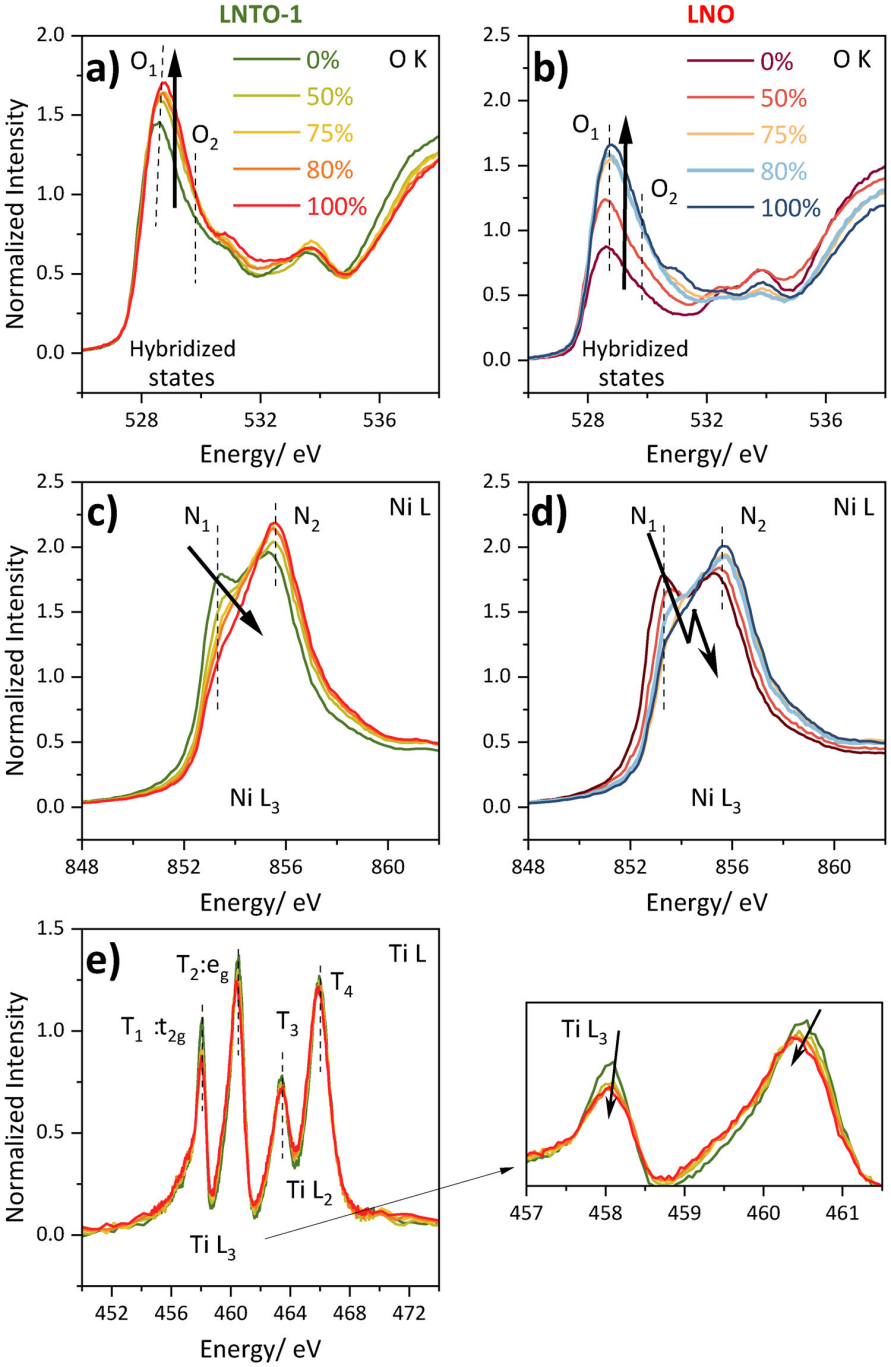
Near‐edge X‐ray absorption fine structure (NEXAFS) technique in the soft X‐ray range demonstrates that Ti substitution stabilizes the electronic structure of LNO. NEXAFS data of cycled samples (LNTO‐1 and LNO) on O K (a,b), Ni L (c,d), and Ti L (e) edges with 0%, 50%, 75%, 80%, and 100% states of charge (SOCs) in fluorescence yield. These data were collected at the KIT light source. To verify the results, cycled LNTO‐1 samples were re‐measured at PTB in Berlin, confirming the same trend, as shown in Figure S17.

During the first cycle, both LNO and LNTO‐1 exhibit an increasing degree of hybridization between *TM* and O orbitals with increasing SOC, as evident by the rising intensity of the O_1_ and O_2_ peaks in Figure 6b. The Ni L edge spectra (Figure 6c,d; Figure S18b) display distinct features at the L_3_ edge (∼851–860 eV) and the L_2_ edge (∼867–877 eV). The L_3_ edge features two main peaks, N_1_ and N_2_, corresponding to an excitation into the unoccupied Ni 3d states from Ni 2p_3/2 core level_ [48, 53, 58]. For simplicity, the N_1_ peak (∼854 eV) is assigned to Ni^2^^+^, while the N_2_ peak (∼873 eV) is associated with Ni^3+^ [56, 62]. However, in the Ni L edge (Figure 6c,d), the oxidation behavior of Ni differs between the two materials. In LNTO‐1, Ni^2^^+^ is continuously oxidized to Ni^3^^+^ throughout cycling. In contrast, in LNO, Ni^3^^+^ initially increases while Ni^2^^+^ decreases from 0% to 75% SOC. Between 75% and 80% SOC, a slight reduction of Ni^3^^+^ back to Ni^2^^+^ occurs, after which the previous oxidation trend (Ni^3+^↑ and Ni^2+^↓) resumes until 100% SOC. This trend is confirmed by changes in the O K edge; e.g., the 75% and 80% SOC spectra of LNO lie on top of each other. As reported in the literature [62, 63], this phenomenon is likely associated with anionic redox, finally leading to oxygen release, which partially reduces Ni^3+^ back to Ni^2+^. It is known to be highly irreversible and is observed specifically during the H2–H3 phase transition in LNO, previously identified as highly detrimental to cycling performance. This phenomenon is barely observed in LNTO‐1, further supporting that Ti substitution enhances the structural stability of the oxygen framework compared to LNO. Ti^4+^ also exerts an electron‐withdrawing effect on the oxygen lattice (inductive effect), as evidenced by the enhanced O K pre‐edge of pristine LNTO‐1 compared to pristine LNO (Figure 6a,b). This effect is expected to be particularly pronounced for Ti located within the *TM* layer (3a sites), where strong in‐plane *TM*–O orbital hybridization arises from the edge‐sharing *TM*O_6_ network characteristic of layered $R\bar{3}m$ structures, whereas the interlayer electronic coupling involving Ti in 3b sites is comparatively weaker [64].

Similar to the Ni L edge, the Ti L edge spectra (Figure 6e) are characterized by the L_3_(∼455‐461 eV, T_1_ and T_2_ peaks) and L_2_ edge (∼462‐465 eV, T_3_ and T_4_). These features are separated by ∼4 eV due to spin‐orbit coupling of the Ti 2p atomic orbitals [48, 53, 65, 66, 67, 68]. Further splitting of the L_3_ and L_2_ transitions into doublet features arises from symmetry effects within the crystal field, as well as covalent or ionic interactions between the ligand and the *TM* center [48, 53]. In Ti(IV) oxides, these doublets are typically attributed to the *t*_2g_ and *e*_g_ manifolds, which are characteristic of octahedral (O_h_ symmetry) or distorted octahedral symmetry [59, 65, 69].

In the case of TiO_2_, either in rutile or anatase, the TiO_6_ octahedra are tetragonally distorted, leading to a reduction in symmetry from O_h_ to D_2h_ (rutile) or D_2d_ (anatase) [70]. These TiO_2_ phases exhibit spectral features similar to those observed in LNTO‐1 (Figure 6e; Figure S18c), suggesting that Ti in LNTO‐1 may exist within a locally distorted coordination environment. It should be noted that such local structural asymmetry is fundamentally different from the Jahn–Teller distortion associated with the interconnected NiO_6_ network: Ti^4+^ possesses a d^0^ electronic configuration and thus it is not expected to undergo Jahn–Teller distortions. In both the pristine sample (Figure S18c) and cycled samples (Figure 6e), the Ti L‐edge consistently displays four well‐defined peaks at similar positions, indicating that this locally slightly distorted environment persists throughout the electrochemical cycling process. However, with increasing SOC, the intensity decreases, and the peaks shift slightly toward lower energies, which may result from changes in the Ti─O bond length or the distorted environment during delithiation. At high SOCs, the electron‐withdrawing effect of Ti on the oxygen lattice may become especially stabilizing [60, 62], as it weakens the inductive electron‐withdrawing effect of highly oxidized Ni species on oxygen, thereby reducing the susceptibility of lattice oxygen toward irreversible oxidation and oxygen release. Furthermore, because Ti, similar to Co [41, 56], remains largely redox‐inactive at high SOCs, it helps maintain a relatively stable local coordination environment and buffers structural perturbations arising from the changing oxidation states of neighboring Ni ions.

To investigate the long‐term stabilization effect of Ti substitution, NEXAFS measurements at the O K, Ni L, and Ti L edges were carried out for LNO and LNTO‐1 after 1 and 100 cycles Figures S18 and S19. The O K‐edge spectra indicate that both materials experience a reduction of the hybridization‐related O_1_/O_2_ pre‐edge peaks during cycling, accompanied by the emergence of Hubbard‐band‐related O_3_/O_4_ features associated with structural degradation [71]. These spectral changes reflect oxygen‐depletion‐induced structural reconstruction, in which transition metal migration into Li 3b sites and/or transition metal dissolution promotes the transformation of the original rhombohedral layered oxide into spinel‐ or rock‐salt‐like domains with increased Mott–Hubbard character and structural disorder [56, 62, 72, 73, 74]. These transformations are particularly pronounced at the grain boundaries and progressively propagate into the bulk structure, as evidenced by the less pronounced changes in the bulk‐sensitive FY spectra (∼several hundred nm probing depth, Figure S18) compared with the surface‐sensitive TEY spectra (∼2–5 nm probing depth, Figure S19). This evolution is generally detrimental, as it correlates with capacity fading and reduced electrochemical reversibility during prolonged cycling [72].

The observed changes are significantly less pronounced in LNTO‐1, suggesting that Ti substitution helps stabilize the oxygen framework and suppresses oxygen depletion during long‐term cycling. The Ni L‐edge spectra further reveal that LNTO‐1 maintains a largely reversible Ni redox behavior after 100 cycles, whereas LNO exhibits stronger Ni^2+^ features, consistent with the formation of spinel/rock‐salt‐like phases revealing strong Hubbard peaks in the O K edge (peak O_3_ and O_4_, Figure S19a). After 100 cycles, the splitting of the Ti L_3_ and L_2_ edges remains largely unchanged, indicating that Ti maintains its coordination environment and associated crystal field splitting (Figure S19c). *TM* dissolution is typically accompanied by changes in the local electronic environment and oxidation state of the metals, which are readily detectable by NEXAFS, particularly in the surface‐sensitive TEY mode, as previously reported for Mn and Co dissolution in layered oxides [75]. Thus, the stable Ti signal observed at the surface upon the 100^th^ cycle (Figure S19c) supports the role of Ti in stabilizing the surface lattice structure by an electron‐withdrawing effect. Surface‐sensitive TEY‐NEXAFS measurements also confirm that LNTO‐1 retains surface‐active oxygen species and partially preserved Ni^3+^ after prolonged cycling, while LNO undergoes severe surface passivation toward a NiO‐like rock‐salt structure (Figure S19b, increasing Ni^2+^ peaks N1). Overall, these results demonstrate that Ti substitution enhances both bulk and surface structural stability, suppresses irreversible oxygen oxidation and structural degradation, and thereby improves the long‐term cycling stability of LNTO‐1 compared to pristine LNO.

Based on the above analysis, LNTO‐1 demonstrates superior long‐term cycling stability compared to LNO, primarily attributed to Ti substitution within the rhombohedral structure not only in the bulk but also on the surface. This substitution helps suppress phase transitions, eliminate Jahn–Teller distortion, and mitigate structural degradation both in the bulk and on the surface during cycling.

## 3 Conclusion

This study presents Ti substitution as a promising strategy to stabilize Co‐free high‐Ni layered oxide cathodes for LIBs. Phase‐pure LNTOs were successfully prepared, with LNTO‐1 showing a high initial discharge capacity of 197.5 mAh g^−^^1^ at 0.05 C and superior cycling stability, retaining 80% of its capacity after 431 cycles at 0.5 C and thus clearly outperforming pristine LiNiO_2_. *Operando* synchrotron XRD reveals that introducing Ti suppresses all phase transitions (H1‐M, M‐H2, and H2‐H3), significantly reducing mechanical strain and structural degradation. EXAFS analyses show the suppression of Jahn–Teller distortions in the NiO_6_ octahedra of LNTO‐1. This stabilization effect is attributed to TiO_6_ octahedra acting as compensatory structural units that accommodate local lattice strain within the interconnected NiO_6_ framework, leading to improved structural reversibility. In addition to this pillar‐like local structural stabilization, theoretical calculations reveal that Ti substitution enlarges the Ni 3d‐O 2p energy separation, indicating an inductive electronic effect that weakens Ni─O covalency and reduces the driving force for Jahn–Teller distortion. Therefore, the improved electrochemical performance of LNTO‐1 originates from the synergistic structural and electronic stabilization effects of TiO_6_ units. These findings clarify the mechanistic role of Ti substitution beyond empirical performance improvement and provide guidance for the rational design of Co‐free high‐Ni layered oxide cathodes for next‐generation lithium‐ion batteries.

## 4 Experimental Section

### 4.1 Material Synthesis

The precursors of LNO were synthesized using a lab‐scale setup. A 1.5 M aqueous solution of metal ions consisting of NiSO_4_·6H_2_O (Acros Organics, purity: 99%), a 12 wt.% ammonia solution (Acros Organics, purity: ≥99%), and a 4.875 M sodium hydroxide solution (Fischer Chemical, purity: 99.44%) were separately and simultaneously pumped into the flask, respectively. The pH of the mixture was controlled at ∼11 with the temperature maintained at 60°C and a stirring speed of 500 rpm. Following co‐precipitation, the precipitates were thoroughly washed with distilled water in a Büchner funnel with Büchner flask suction until the pH reached 7. The precipitates were then dried overnight at 80°C. To obtain the final rhombohedral lithium transition metal oxides (LiNiO_2_), the precursor was mixed with LiOH∙H_2_O (Fischer Chemical, purity: ≥ 99%) in a molar ratio of 1:1.03 and preheated to 500°C for 4 h with a heating rate of 2°C min^−1^ and later calcined at 680°C for 10 h with O_2_ flow. The LNTO‐1 and LNTO‐2 samples were synthesized by introducing a transition‐metal solution containing Ni and Ti ions during the co‐precipitation process, followed by lithiation and calcination at 800°C. Ti was introduced using TiOSO_4_ as the Ti source (Acros Organics, purity ≥ 99%). The nominal Ti feeding ratios were 0.10 and 0.20 for LNTO‐1 and LNTO‐2, respectively, while the final Ti contents in the calcined products, as determined by ICP‐OES, were *x* = 0.07 and 0.14, respectively. Therefore, the compositions of LNTO‐1 and LNTO‐2 are denoted as LiNi_0.93_Ti_0.07_O_2_ and LiNi_0.86_Ti_0.14_O_2_ throughout this work. This synthesis method was optimized in a previous publication [76].

#### 4.1.1 Sample Characterization: Scanning Electron Microscopy (SEM)

The morphology of the synthesized precursors and final products was examined using scanning electron microscopy (SEM, Carl Zeiss AURIGA, Carl Zeiss Microscopy GmbH) at an accelerating voltage of 3.0 kV and a working distance of 3 mm.

#### 4.1.2 Energy Dispersive X‐Ray Spectroscopy (SEM‐EDX)

For mapping measurements, an accelerating voltage of 3.0 kV and a working distance of 5 mm were employed. Energy dispersive X‐ray spectroscopy was conducted using an X‐MAX 80 mm^2^ detector to analyze the elemental composition of the samples. The acquired spectra were analyzed using the INCA software developed by Oxford Instruments.

### 4.2 Synchrotron X‐Ray Powder Diffraction (SXPD)

The phase purity of the various cathodes was confirmed through powder diffraction measurements at DESY P02.1 (λ = 0.20728Å). Additionally, the *operando* cycling processes of LNO and LNTO‐1 were investigated at the same beamline with self‐designed cell bodies. These cells ensured homogeneous pressure on the working electrodes. Details can be found in Ref. [77].

#### 4.2.1 Particle Size Analyzer (PSA)

A particle size analysis was performed with a CILAS Particle size analyzer.

#### 4.2.2 Inductively Coupled Plasma Optical Emission Spectroscopy (ICP‐OES)

The measurements were performed using an ARCOS spectrometer from SPECTRO Analytical Instruments Company (Germany), with axial plasma viewing, and a standard Fassel‐type torch was utilized. During microwave digestion, LNTO‐1 and LNTO‐2 initially showed slight turbidity with greenish residues. After standing for a short period, the residues gradually disappeared, resulting in clear solutions. These clear digested solutions were then used for ICP‐OES measurements to determine the total elemental compositions.

#### 4.2.3 Transmission Electron Microscopy (TEM)

TEM images were obtained at 300 kV from a high‐resolution field‐emission transmission electron microscope (FEI Tecnai G2 F30).

### 4.3 X‐Ray Photoelectron Spectroscopy (XPS)

The cycled electrode was removed from the respective cell in a glovebox, washed with DMC and dried under reduced pressure. The sample was then transported in a sealed vessel to a glovebox connected to an Axis Ultra DLD spectrometer (Kratos), where it was held in the instrument´s ultra‐high vacuum chamber for 12 h to completely remove volatile species. The measurement was conducted under ultra‐high vacuum (<10−8 mbar) using a monochromatic Al Kα source (hν = 1486.6 eV) with 10 mA emission current, and an accelerating voltage of 12 kV, at an 0° angle of emission. In order to compensate for charging of the sample, a charge neutralizer was used. Five core spectra each for Ni 2p, F 1s, Ti 2p, O 1s, C 1s, and Li 1s hyperregion were acquired using a pass energy of 20 eV, and a “hybrid” lens mode on an analysis area of 700 µm x 300 µm for two different spots. Sputter depth profiling was carried out using monochromatic argon. An accelerating voltage of 500 V and an extractor current of 15 µA were used. The diameter of the sputter crater is approximately 2 mm. Survey spectra at a pass energy of 160 eV and core spectra were acquired again after sputtering times of 60, 120, 180, 240, 300, 360, 490, 600, and 1200 s. The resulting data were evaluated with CasaXPS (v2.3.25, Casa Software) [78]. The five individual measurements for each core spectrum were summed. The data were then internally calibrated to the positions of the M‐F/Li‐F peaks at a binding energy of 685 eV.

### 4.4 Near Edge X‐Ray Absorption Fine Structures Spectroscopy (NEXAFS)

To ensure uniform performance, 10‐coin cells were assembled for the first cycle. Each electrode's cycling performance was evaluated, and only the most representative electrodes were selected for NEXAFS measurements. The electrodes were fully charged and then discharged to a specific state of charge (SOC) using a constant current of C/20. The SOC was determined based on the specific capacity of the previous cycle. Subsequently, the cells were disassembled within the glovebox. Following disassembly, the electrodes were vacuum‐dried overnight in a Buechi Oven. They were then mounted onto the sample plate and transferred to the beamline using a transfer case to mitigate air contamination. NEXAFS data were collected at IQMT's soft X‐ray beamline WERA at the Karlsruhe synchrotron light source KARA (Germany) and PTB (Physikalisch‐Technische Bundesanstalt) at the PGM beamline in the PTB laboratory at BESSY II in Berlin, Germany. NEXAFS measurements at Ni L_2,3_, Ti L_2,3_, and O K were carried out simultaneously in fluorescence yield (FY) and total electron yield (TEY) detection mode. The photon‐energy step size in the spectra was set to 0.2–0.4 eV.

### 4.5 X‐Ray Absorption Spectroscopy (XANES and EXAFS)

The *operando* XAS spectra on LNO and LNTO‐1 were recorded at the B18 beamline [42, 79, 80] of Diamond Light Source, using the same cell body as in the *operando* SXPD experiments to ensure data comparison. Ni K‐edge spectra were collected during cycling in transmission mode, while Ti K‐edge spectra were measured *ex situ* at specific states of charge: 0%, 25%, 50%, 75%, and 100% in fluorescence mode using an ME‐4 Silicon Drift Fluorescence Detector. Measurements have been made using a Si(111) crystal cut of the monochromator using Pt‐coated collimating, focusing, and harmonic‐rejection mirrors (HRMs). The dedicated pair of harmonic rejection mirrors was used to eliminate higher harmonics. The hard X‐ray absorption spectroscopy (XAS) data were processed using the Athena program of the Demeter software package [43]. The energy scale was calibrated by applying the energy shift determined from simultaneously measured reference foils. Background subtraction and normalization were performed using the same procedure for all absorption edges. The EXAFS spectra, χ(k), were extracted and Fourier transformed from *k*‐space to *R*‐space using Athena. Quantitative fitting of the EXAFS data was carried out with the Artemis program of the Demeter package to determine the Ni─O, Ni─TM, Ti─O, and Ti─TM bond distances.

### 4.6 Electrochemical Characterization

Electrode preparations were performed using 80 wt.% of the cathode materials, 10 wt.% C65 conductive carbon (Super C65, Imerys Graphite & Carbon), 10 wt.% polyvinylidene fluoride (PVDF, Kynar Flex), and N‐methylpyrrolidone (NMP, Sigma–Aldrich, 99.5%) as processing solvent. The electrode paste was cast onto an Al‐foil (20 µm thickness, Goodfellow) with a doctor blade (gap height 200 µm, ZAF 2010, Zehntner). The wet film was dried at 80°C overnight in an oven. The 12 mm diameter samples were punched out of the dried electrodes and assembled in a dry room into 2032‐coin cells with two layers of Celgard2500 (16 mm Ø), 36 µL LP572 (1 M LiPF_6_ in ethylene carbonate (EC): ethyl methyl carbonate (EMC), 3:7 by weight, with 2 wt.% vinylene carbonate (VC), BASF, battery grade) and metallic lithium as a counter electrode (15 mm Ø, Albemarle, battery grade). The as‐prepared samples' rate performance, cycling stability, and galvanostatic intermittent titration technique (GITT) are measured using a Maccor Series 4000 automated test system at 20°C, applying a voltage of 3.0–4.3 V. Each electrochemical investigation involved three‐coin cells. The electrodes have an average mass loading of ∼6 mg cm^−2^. In specific current and capacity calculations, an active material mass value of ∼4.8 mg cm^−2^ was utilized. A nominal capacity of 180 mAh g^−1^ was used to determine the C‐rates. Consequently, constant currents of 9, 18, 36, 90, 180, and 360 mA g^−1^ were applied for C‐rates of 0.05, 0.1, 0.2, 0.5, 1, and 2 C, respectively.

### 4.7 Density Functional Theory

Calculations were performed using the projector augmented‐wave (PAW) method [81] as implemented in the Vienna Ab initio Simulation Package (VASP) [82, 83]. The exchange–correlation interactions were treated within the generalized gradient approximation (GGA) [84] using the Perdew–Burke–Ernzerhof (PBE) functional. A plane‐wave energy cutoff of 520 eV was employed for all calculations. The total energy and atomic forces were converged to within 1 × 10^−^^7^ eV and 0.01 eV/Å, respectively. LiNiO_2_ used a 12 × 12 × 2 k‐point mesh centered at the Gamma point. For the calculations of Ti‐doped LiNiO_2_, a 3 × 3 × 1 unit cell was used, with a 2 × 2 × 1 k‐point mesh centered at the Gamma point.

### 4.8 Statistical Analysis

The X‐ray diffraction patterns were analyzed using the software package Fullprof [85, 86]. The Ni/Li disorder was calculated from Rietveld refinements (the refined occupancy of Ni in the Li 3b sites/amount of 3a sites * 100%). The structural input parameters for LNO were obtained from the crystallographic data file [87]. The refinement analysis includes the estimated standard deviations of the lattice parameters, which have been adjusted by multiplying them with the correlated residuals [88, 89]. XRD refinement was used to evaluate possible crystallographic site occupancy.

The EXAFS data are energy calibrated, normalized, and analyzed by ATHENA, and fitted by Artemis [90]. For electrochemical characterization, each evaluation was conducted using the average of three coin cells, with error bars/ standard deviations represented in the corresponding diagrams.

#### 4.8.1 NEXAFS Data Analysis

A NiO reference was used for data energy calibration [91, 92]. The spectra were recorded over a broad photon‐energy range, covering regions well below and well above the absorption features of the samples. The FY signal was normalized following an established procedure: (1) I_0_ normalization: The raw FY signals collected from four fluorescence detectors positioned at different angles were summed. The incident X‐ray intensity, I_0_, was measured using the drain current from a freshly Au‐coated mesh inserted into the beam path upstream of the sample, before the X‐rays impinged on the sample. Dark counts, corresponding to the residual signal recorded with the shutter closed, were subtracted. The normalized FY signal was then obtained by dividing the summed raw FY signal by the corrected incident intensity. (2) Background subtraction: A linear sloping background was removed by fitting a straight line to the flat low‐energy region of the NEXAFS spectrum, where no absorption features are present. (3) Edge‐jump normalization: The spectrum was normalized by setting the flat low‐energy pre‐edge region to zero and the post‐edge region to unity, corresponding to a unit edge jump. The photon‐energy range selected for the post‐edge normalization was 590–605 eV, which lies well above the absorption features. The normalization is not sensitive to the exact choice of this range, as it is sufficiently far above any compound‐specific resonances. The normalization of the TEY signal follows a similar procedure.

## Author Contributions

**Bixian Ying**: conceptualization, investigation, data curation, formal analysis, Writing – original draft, Writing – review and editing, software, validation, visualization. **Verena Naber**: methodology. **Sascha Nowak**: methodology. **Martin Winter**: supervision, funding acquisition, writing – review and editing, project administration, resources. **Karin Kleiner**: funding acquisition, supervision, conceptualization, project administration, writing – review and editing, resources, visualization. **Zhenjie Teng**: investigation, data curation, writing – review and editing, formal analysis, visualization. **Honghong Tian**: investigation. **Alexander Schökel**: methodology. **Iuliia Mikulska**: methodology, writing – review and editing, formal analysis. **Michael Merz**: methodology. **Rommel T. Tolla**: investigation, writing – review and editing. **Katja Frenzel**: methodology. **Yuanming Liu**: methodology. **Lena Mathies**: methodology. **Charlotte von Petersdorff‐campen**: investigation. **Jun Wang**: writing – review and editing. **Adrian Jonas**: methodology, writing – review and editing. **Peter Nagel**: methodology. **Stefan Schuppler**: methodology. **Linus Voigt**: methodology.

## Conflicts of Interest

The authors declare no conflicts of interest.

## Supporting information




**Supporting File**: advs77138‐sup‐0001‐SuppMat.docx.

## Data Availability

The authors have nothing to report.

## References

[1] J. Liu , Z. Bao , Y. Cui , et al., “Pathways for Practical High‐Energy Long‐Cycling Lithium Metal Batteries,” Nature Energy 4, no. 3 (2019): 180–186.

[2] S. Dühnen , J. Betz , M. Kolek , R. Schmuch , M. Winter , and T. Placke , “Toward Green Battery Cells: Perspective on Materials and Technologies,” Small Methods 4 (2020): 2000039, 10.1002/smtd.202000039.

[3] V. R. Stamenkovic , D. Strmcnik , P. P. Lopes , and N. M. Markovic , “Energy and Fuels From Electrochemical Interfaces,” Nature Materials 16, no. 1 (2017): 57–69. 10.1038/nmat4738 27994237

[4] R. Schmuch , R. Wagner , G. Hörpel , T. Placke , and M. Winter , “Performance and Cost of Materials for Lithium‐Based Rechargeable Automotive Batteries,” Nature Energy 3, no. 4 (2018): 267–278.

[5] S.‐T. Myung , F. Maglia , K. J. Park , et al., “Nickel‐Rich Layered Cathode Materials for Automotive Lithium‐Ion Batteries: Achievements and Perspectives,” ACS Energy Letters 2, no. 1 (2017): 196–223.

[6] D. Andre , S. J. Kim , P. Lamp , et al., “Future Generations of Cathode Materials: An Automotive Industry Perspective,” Journal of Materials Chemistry A 3, no. 13 (2015): 6709–6732.

[7] S. S. Zhang , “Problems and Their Origins of Ni‐Rich Layered Oxide Cathode Materials,” Energy Storage Materials 24 (2020): 247–254.

[8] J.‐M. Tarascon and M. Armand , “Issues and Challenges Facing Rechargeable Lithium Batteries,” Nature 414, no. 6861 (2001): 359–367. 10.1038/35104644 11713543

[9] M. S. Whittingham , “Lithium Batteries and Cathode Materials,” Chemical Reviews 104, no. 10 (2004): 4271–4302. 10.1021/cr020731c 15669156

[10] W. Liu , P. Oh , X. Liu , et al., “Nickel‐Rich Layered Lithium Transition‐Metal Oxide for High‐Energy Lithium‐Ion Batteries,” Angewandte Chemie International Edition 54, no. 15 (2015): 4440–4457. 10.1002/anie.201409262 25801735

[11] S. Yin , W. Deng , J. Chen , et al., “Fundamental and Solutions of Microcrack in Ni‐Rich Layered Oxide Cathode Materials of Lithium‐Ion Batteries,” Nano Energy 83 (2021): 105854, 10.1016/j.nanoen.2021.105854.

[12] H. Zhao , W. Y. A. Lam , L. Sheng , et al., “Cobalt‐Free Cathode Materials: Families and Their Prospects,” Advanced Energy Materials 12, no. 16 (2022): 2103894.

[13] J. Reed and G. Ceder , “Role of Electronic Structure in the Susceptibility of Metastable Transition‐Metal Oxide Structures to Transformation,” Chemical Reviews 104, no. 10 (2004): 4513–4534. 10.1021/cr020733x 15669161

[14] Y. Luo , H. Wei , L. Tang , et al., “Nickel‐Rich and Cobalt‐Free Layered Oxide Cathode Materials for Lithium Ion Batteries,” Energy Storage Materials 50 (2022): 274–307.

[15] N. Muralidharan , E. C. Self , J. Nanda , et al., “Next‐Generation Cobalt‐Free Cathodes—A Prospective Solution to the Battery Industry's Cobalt Problem,” Advanced Energy Materials 12 (2022): 33–53.

[16] J. Kim and K. Amine , “The Effect of Tetravalent Titanium Substitution in LiNi_1−x_TixO_2_ (0.025⩽ x⩽ 0.2) System,” Electrochemistry Communications 3, no. 2 (2001): 52–55.

[17] M. Song , C. K. Park , S. D. Yoon , H. R. Park , and D. R. Mumm , “Electrochemical Properties of LiNi_1−y_MyO_2_ (M = Ni, Ga, Al and/or Ti) Cathodes,” Ceramics International 35, no. 3 (2009): 1145–1150.

[18] S. Sicolo , M. Mock , M. Bianchini , and K. Albe , “And Yet It Moves: LiNiO_2_, a Dynamic Jahn–Teller System,” Chemistry of Materials 32, no. 23 (2020): 10096–10103.

[19] A. R. Genreith‐Schriever , A. Alexiu , G. S. Phillips , et al., “Jahn–Teller Distortions and Phase Transitions in LiNiO_2_: Insights From ab Initio Molecular Dynamics and Variable‐Temperature X‐Ray Diffraction,” Chemistry of Materials 36, no. 5 (2024): 2289–2303. 10.1021/acs.chemmater.3c02413 PMC10938510 38495898

[20] L. Mu , R. Zhang , W. H. Kan , et al., “Dopant Distribution in Co‐Free High‐Energy Layered Cathode Materials,” Chemistry of Materials 31, no. 23 (2019): 9769–9776.

[21] C. S. Yoon , M. J. Choi , D. W. Jun , et al., “Cation Ordering of Zr‐Doped LiNiO_2_ Cathode for Lithium‐Ion Batteries,” Chemistry of Materials 30, no. 5 (2018): 1808–1814.

[22] G.‐T. Park , S. B. Kim , B. Namkoong , et al., “A New Ternary Co‐Free Layered Cathode, Li[Ni_1−x−y_TixAly]O_2_, for High‐Energy Lithium‐Ion Batteries,” Materials Today 71 (2023): 38–49.

[23] D. Kong , J. Hu , Z. Chen , et al., “Ti‐Gradient Doping to Stabilize Layered Surface Structure for High Performance High‐Ni Oxide Cathode of Li‐Ion Battery,” Advanced Energy Materials 9, no. 41 (2019): 1901756.

[24] H.‐H. Ryu , G.‐T. Park , C. S. Yoon , and Y.‐K. Sun , “Suppressing Detrimental Phase Transitions via Tungsten Doping of LiNiO_2_ Cathode for Next‐Generation Lithium‐Ion Batteries,” Journal of Materials Chemistry A 7, no. 31 (2019): 18580–18588.

[25] H. Li , P. Zhou , F. Liu , H. Li , F. Cheng , and J. Chen , “Stabilizing Nickel‐Rich Layered Oxide Cathodes by Magnesium Doping for Rechargeable Lithium‐Ion Batteries,” Chemical Science 10, no. 5 (2019): 1374–1379. 10.1039/c8sc03385d PMC6354825 30809353

[26] Y. Wang , Z. Feng , P. Cui , et al., “Pillar‐Beam Structures Prevent Layered Cathode Materials From Destructive Phase Transitions,” Nature Communications 12, no. 1 (2021): 13. 10.1038/s41467-020-20169-1 PMC7782780 33397895

[27] J. Li , G. Liang , W. Zheng , et al., “Structure Flexibility Enabled by Surface High‐Concentration Titanium Doping for Durable Lithium‐Ion Battery Cathodes,” Journal of the American Chemical Society 147, no. 22 (2025): 18606–18617. 10.1021/jacs.5c00789 40389807

[28] R. Shi , N. Zheng , H. Ji , et al., “Homogeneous Repair of Highly Degraded Ni‐Rich Cathode Material With Spent Lithium Anode,” Advanced Materials 36, no. 13 (2024): 2311553. 10.1002/adma.202311553 38124361

[29] J. Ma , J. Wang , K. Jia , et al., “Adaptable Eutectic Salt for the Direct Recycling of Highly Degraded Layer Cathodes,” Journal of the American Chemical Society 144, no. 44 (2022): 20306–20314. 10.1021/jacs.2c07860 36228162

[30] K. Jia , J. Wang , Z. Zhuang , et al., “Topotactic Transformation of Surface Structure Enabling Direct Regeneration of Spent Lithium‐Ion Battery Cathodes,” Journal of the American Chemical Society 145, no. 13 (2023): 7288–7300. 10.1021/jacs.2c13151 36876987

[31] C. Zhao , Z. Yang , X. Zhou , et al., “Recent Progress on Electrolyte Boosting Initial Coulombic Efficiency in Lithium‐Ion Batteries,” Advanced Functional Materials 31, no. 5 (2024): 2303457.

[32] Z. Deng and A. Manthiram , “Influence of Cationic Substitutions on the Oxygen Loss and Reversible Capacity of Lithium‐Rich Layered Oxide Cathodes,” Journal of Physical Chemistry C 115, no. 14 (2011): 7097–7103.

[33] L. de Biasi , A. Schiele , M. Roca‐Ayats , et al., “Phase Transformation Behavior and Stability of LiNiO_2_ Cathode Material for Li‐Ion Batteries Obtained From In Situ Gas Analysis and Operando X‐Ray Diffraction,” ChemSusChem 12, no. 10 (2019): 2240–2250. 10.1002/cssc.201900032 30839177

[34] H. Li , W. Hua , X. Liu‐Théato , et al., “New Insights Into Lithium Hopping and Ordering in LiNiO_2_ Cathodes During Li (de) Intercalation,” Chemistry of Materials 33 (2021): 9546–9559.

[35] M. Bianchini , A. Schiele , S. Schweidler , et al., “From LiNiO_2_ to Li_2_NiO_3_: Synthesis, Structures and Electrochemical Mechanisms in Li‐Rich Nickel Oxides,” Chemistry of Materials 32, no. 21 (2020): 9211–9227.

[36] E. Fermi , Thermodynamics (Courier Corporation, 2012).

[37] H. B. Callen , Thermodynamics and An Introduction to Thermostatistics (John Wiley& Sons, 1993).

[38] T. Ohzuku , A. Ueda , and M. Nagayama , “Electrochemistry and Structural Chemistry of LiNiO_2_(R3m) for 4 Volt Secondary Lithium Cells,” Journal of the Electrochemical Society 140, no. 7 (1993): 1862.

[39] P. Zhao , L. Chen , L. Yao , et al., “Compositional Gradient Design and Microstructural Engineering Enable Ultra‐Stable Quinary Ni‐Rich Cathodes in High‐Voltage Pouch Cells,” Science Bulletin 71, no. 5 (2026): 1113. 10.1016/j.scib.2025.12.007 41558939

[40] C. Zhao , C. Wang , X. Liu , et al., “Suppressing Strain Propagation in Ultrahigh‐Ni Cathodes During Fast Charging via Epitaxial Entropy‐Assisted Coating,” Nature Energy 9, no. 3 (2024): 345–356.

[41] W. Guo , H. Yu , M. Wang , et al., “Compositional Gradient Design of Ni‐Rich Co‐Poor Cathodes Enhanced Cyclability and Safety in High‐Voltage Li‐Ion Batteries,” ACS nano 19, no. 8 (2025): 8371–8380. 10.1021/acsnano.5c00974 39973286

[42] C. T. Chantler , G. Bunker , P. D'Angelo , and S. Diaz‐Moreno , “X‐Ray Absorption Spectroscopy,” Nature Reviews Methods Primers 4, no. 1 (2024): 1–25.

[43] B. Ravel and M. Newville , “ATHENA, ARTEMIS, HEPHAESTUS: Data Analysis for X‐Ray Absorption Spectroscopy Using IFEFFIT,” Synchrotron Radiation 12 (2005): 537–541. 10.1107/S0909049505012719 15968136

[44] A. Rougier , C. Delmas , and A. V. Chadwick , “Non‐Cooperative Jahn‐Teller Effect in LiNiO_2_: An EXAFS Study,” Solid State Communications 94, no. 2 (1995): 123–127.

[45] M. Bianchini , M. Roca‐Ayats , P. Hartmann , T. Brezesinski , and J. Janek , “There and Back Again—The Journey of LiNiO_2_ as a Cathode Active Material,” Angewandte Chemie International Edition 58, no. 31 (2019): 10434–10458. 10.1002/anie.201812472 30537189

[46] I. Nakai , K. Takahashi , Y. Shiraishi , T. Nakagome , and F. Nishikawa , “Study of the Jahn–Teller Distortion in LiNiO_2_, a Cathode Material in a Rechargeable Lithium Battery, by In Situ X‐Ray Absorption Fine Structure Analysis,” Journal of Solid State Chemistry 140, no. 1 (1998): 145–148.

[47] F. De Groot , “High‐Resolution X‐Ray Emission and X‐Ray Absorption Spectroscopy,” Chemical Reviews 101, no. 6 (2001): 1779–1808. 10.1021/cr9900681 11709999

[48] F. De Groot and A. Kotani , Core Level Spectroscopy of Solids (CRC Press, 2008).

[49] Y. W. Tsai , B. J. Hwang , G. Ceder , H. S. Sheu , D. G. Liu , and J. F. Lee , “In‐Situ X‐Ray Absorption Spectroscopic Study on Variation of Electronic Transitions and Local Structure of LiNi_1/3_Co_1/3_Mn_1/3_O_2_ Cathode Material During Electrochemical Cycling,” Chemistry of Materials 17, no. 12 (2005): 3191–3199.

[50] P.‐Y. Liao , J.‐G. Duh , and J.‐F. Lee , “Valence Change and Local Structure During Cycling of Layer‐Structured Cathode Materials,” Journal of Power Sources 189, no. 1 (2009): 9–15.

[51] C. F. Petersburg , Z. Li , N. A. Chernova , M. S. Whittingham , and F. M. Alamgir , “Oxygen and Transition Metal Involvement in the Charge Compensation Mechanism of LiNi_1/3_Mn_1/3_Co_1/3_O_2_ Cathodes,” Journal of Materials Chemistry 22, no. 37 (2012): 19993–20000.

[52] A. O. Kondrakov , H. Geßwein , K. Galdina , et al., “Charge‐Transfer‐Induced Lattice Collapse in Ni‐Rich NCM Cathode Materials During Delithiation,” Journal of Physical Chemistry C 121, no. 44 (2017): 24381–24388.

[53] G. S. Henderson , F. M. De Groot , and B. J. Moulton , “X‐Ray Absorption Near‐Edge Structure (XANES) Spectroscopy,” Reviews in Mineralogy Geochemistry 78, no. 1 (2014): 75–138.

[54] F. Farges , G. E. Brown , and J. Rehr , “Ti K‐Edge XANES Studies of Ti Coordination and Disorder in Oxide Compounds: Comparison Between Theory and Experiment,” Physical Review B 56, no. 4 (1997): 1809.

[55] L. X. Chen , T. Rajh , Z. Wang , and M. C. Thurnauer , “XAFS Studies of Surface Structures of TiO_2_ Nanoparticles and Photocatalytic Reduction of Metal Ions,” Journal of Physical Chemistry B 101, no. 50 (1997): 10688–10697.

[56] K. Kleiner , C. A. Murray , C. Grosu , et al., “On the Origin of Reversible and Irreversible Reactions in LiNixCo_(1−x)/2_Mn_(1−x)_2O_2_ ,” Journal of the Electrochemical Society 168, no. 12 (2021): 120533.

[57] K. Kleiner , B. Strehle , A. R. Baker , et al., “Origin of High Capacity and Poor Cycling Stability of Li‐Rich Layered Oxides: A Long‐Duration In Situ Synchrotron Powder Diffraction Study,” Chemistry of Materials 30, no. 11 (2018): 3656–3667.

[58] F. M. F. de Groot , X‐ray Absorption of Transition Metal Oxides (Radboud Repository, 1991)1–17.

[59] J. Stöhr , NEXAFS Spectroscope (Springer Science & Business Media, 2013).

[60] H. Yu , J. Cheng , H. Zhu , et al., “Reversible Configurations of 3‐Coordinate and 4‐Coordinate Boron Stabilize Ultrahigh‐Ni Cathodes With Superior Cycling Stability for Practical Li‐ion Batteries,” Advanced Materials 37, no. 1 (2025): 2412360. 10.1002/adma.202412360 39473297

[61] F. Kong , C. Liang , R. C. Longo , et al., “Conflicting Roles of Anion Doping on the Electrochemical Performance of Li‐Ion Battery Cathode Materials,” Chemistry of Materials 28, no. 19 (2016): 6942–6952.

[62] B. Ying , Z. Teng , A. Senyshyn , et al., “Insights Into Homogeneous Bulk Boron Doping at the Tetrahedral Site of NCM811 Cathode Materials: Structure Stabilization by Inductive Effect on TM‐O‐B Bonds,” Small 21 (2025): 2409743. 10.1002/smll.202409743 PMC11878253 39828621

[63] M. Saubanère , E. McCalla , J.‐M. Tarascon , and M.‐L. Doublet , “The Intriguing Question of Anionic Redox in High‐Energy Density Cathodes for Li‐Ion Batteries,” Energy & Environmental Science 9 (2016): 984–991.

[64] M. Okubo and A. Yamada , “Molecular Orbital Principles of Oxygen‐Redox Battery Electrodes,” ACS Applied Materials & Interfaces 9, no. 42 (2017): 36463–36472. 10.1021/acsami.7b09835 29016101

[65] F. De Groot , J. Fuggle , B. Thole , and G. Sawatzky , “3 X‐Ray‐Absorption Edges of d 0 Compounds: K^+^, Ca^2+^, Sc^3+^, and Ti^4+^ in O h (Octahedral) Symmetry,” Physical Review B 2, no. 2 (1990): 928. 10.1103/physrevb.41.928 9993787

[66] T. Kroll , E. I. Solomon , and F. M. de Groot , “An Analysis of Ti L‐Edge Absorption Spectra,” Journal of Physical Chemistry B 119, no. 43 (2015): 13852–13858. 10.1021/acs.jpcb.5b04133 PMC4779055 26226507

[67] F. M. F. De Groot , M. O. Figueiredo , M. J. Basto , M. Abbate , H. Petersen , and J. C. Fuggle , “2p X‐Ray Absorption of Titanium in Minerals,” Physics Chemistry of Minerals 19 (1992): 140–147.

[68] F. M. de Groot , J. Fuggle , B. Thole , and G. Sawatzky , “2p X‐Ray Absorption of 3d Transition‐Metal Compounds: An Atomic Multiplet Description Including the Crystal Field,” Physical Review B 42, no. 9 (1990): 5459. 10.1103/physrevb.42.5459 9996129

[69] J. G. Chen , “NEXAFS Investigations of Transition Metal Oxides, Nitrides, Carbides, Sulfides and Other Interstitial Compounds,” Surface Science Reports 30, no. 1–3 (1997): 1–152.

[70] E. Stoyanov , F. Langenhorst , and S. Neumann , “The Effect of Valence State and Site Geometry on Ti L 3, 2 and OK Electron Energy‐Loss Spectra of TixOy Phases,” American Mineralogist 92, no. 4 (2007): 577–586.

[71] K. Kleiner , J. Melke , M. Merz , et al., “Unraveling the Degradation Process of LiNi_0.8_Co_0.15_Al_0.05_O_2_ Electrodes in Commercial Lithium Ion Batteries by Electronic Structure Investigations,” ACS Applied Materials & Interfaces 7, no. 35 (2015): 19589–19600. 10.1021/acsami.5b03191 26281920

[72] J. Van Elp , H. Eskes , P. Kuiper , and G. Sawatzky , “Electronic Structure of Li‐Doped NiO,” Physical Review B 45, no. 4 (1992): 1612. 10.1103/physrevb.45.1612 10001659

[73] J. Van Elp , B. Searle , G. Sawatzky , and M. Sacchi , “Ligand Hole Induced Symmetry Mixing of d8 States in LixNi_1−x_O, as Observed in Ni 2p X‐Ray Absorption Spectroscopy,” Solid State Communications 80, no. 1 (1991): 67–71.

[74] B. Strehle , K. Kleiner , R. Jung , et al., “The Role of Oxygen Release From Li‐and Mn‐Rich Layered Oxides During the First Cycles Investigated by On‐Line Electrochemical Mass Spectrometry,” Journal of Electrochemical Society 164, no. 2 (2017): A400–A406.

[75] M. Merz , B. Ying , P. Nagel , S. Schuppler , K. Kleiner , et al., “Reversible and Irreversible Redox Processes in Li‐Rich Layered Oxides,” Chemistry of Materials 33, no. 24 (2021): 9534–9545.

[76] B. Ying , J. R. Fitzpatrick , Z. Teng , et al., “Monitoring the Formation of Nickel‐Poor and Nickel‐Rich Oxide Cathode Materials for Lithium‐Ion Batteries With Synchrotron Radiation,” Chemistry of Materials 35, no. 4 (2023): 1514–1526. 10.1021/acs.chemmater.2c02639 PMC9979376 36873624

[77] B. Ying , “Modification of the Electronic Structure of Ni‐rich Layered Oxide as Cathode Materials for Lithium Ion Battery” (PhD thesis, Münster University, 2024).

[78] N. Fairley , V. Fernandez , M. Richard‐Plouet , et al., “Systematic and Collaborative Approach to Problem Solving Using X‐Ray Photoelectron Spectroscopy,” Applied Surface Science Advances 5 (2021): 100112, 10.1016/j.apsadv.2021.100112.

[79] S. Diaz‐Moreno , M. Amboage , M. Basham , et al., “The Spectroscopy Village at Diamond Light Source,” Synchrotron Radiation 25 (2018): 998–1009. 10.1107/S1600577518006173 PMC6038600 29979161

[80] A. J. Dent , G. Cibin , S. Ramos , et al., “B18: A Core XAS Spectroscopy Beamline for Diamond,” Journal of Physics: Conference Series 190 (2009): 012039.

[81] P. Blöchl , “Projector Augmented‐Wave Method,” Physical Review B 50 (1994): 17953. 10.1103/physrevb.50.17953 9976227

[82] G. Kresse and J. Furthmüller , “Efficiency of *Ab‐Initio* Total Energy Calculations for Metals and Semiconductors Using a Plane‐Wave Basis Set,” Computational Materials Science 6 (1996): 15–50.

[83] G. Kresse and J. Furthmüller , “Efficient Iterative Schemes for *ab initio* Total‐Energy Calculations Using a Plane‐Wave Basis Set,” Physical Review B 54 (1996): 11169. 10.1103/physrevb.54.11169 9984901

[84] J. P. Perdew , K. Burke , and M. Ernzerhof , “Generalized Gradient Approximation Made Simple,” Physical Review Letters 77 (1996): 3865. 10.1103/PhysRevLett.77.3865 10062328

[85] J. Rodriguez‐Carvajal and T. Roisnel , “FullProf.98 and WinPLOTR: New Windows 95/NT Applications for Diffraction,” Commission for Powder Diffraction 20 (1998): 5–19.

[86] T. Roisnel , J. Rodríguez‐Carvajal , and J. Gonzalez‐Platas , “WinPLOTR, A Graphic Tool for Powder Diffraction,” Materials Science Forum 378, (2001): 91191.

[87] L.‐J. Li , X.‐H. Li , Z.‐X. Wang , et al., “Synthesis, Structural and Electrochemical Properties of LiNi_0.79_Co_0.1_Mn_0.1_Cr_0.01_O_2_ via Fast Co‐Precipitation,” Journal of Alloys and Compounds 507, no. 1 (2010): 172–177.

[88] J.‐F. Bérar and P. Lelann , “ESD's and Estimated Probable Error Obtained in Rietveld Refinements With Local Correlations,” Journal of Applied Crystallography 24, no. 1 (1991): 1–5.

[89] J. Berar , “Data Optimization and Propagation of Errors in Powder Diffraction,” Accuracy in Powder Diffraction II 846 (1992): 63.

[90] B. Ravel and M. Newville , “Data Analysis for X‐Ray Absorption Spectroscopy Using IFEFFIT,” Synchrotron Radiation 12 (2005): 537–541. 10.1107/S0909049505012719 15968136

[91] M. Merz , P. Nagel , C. Pinta , et al., “X‐ray Absorption and Magnetic Circular Dichroism of LaCoO_3_, La_0.7_Ce_0.3_CoO_3_, and La_0.7_Sr_0.3_CoO_3_ Films: Evidence for Cobalt‐Valence‐Dependent Magnetism,” Physical Review B 82, no. 17 (2010): 174416.

[92] M. Merz , D. Fuchs , A. Assmann , et al., “Spin and Orbital States in Single‐Layered La_2−x_Ca_x_CoO_4_ Studied by Doping‐and Temperature‐Dependent Near‐Edge X‐Ray Absorption Fine Structure,” Physical Review B 84, no. 1 (2011): 014436.

