# Supplementary material for: TiO6 as a Compensatory Structural Unit in Co‐Free, High‐Ni Layered Oxide for Durable Lithium‐Ion Batteries
Source: Adv Sci (Weinh). 2026 Aug 24;13(63):e77138. doi: 10.1002/advs.77138 (PMC13502363; doi:10.1002/advs.77138)
Supplement: Supplementary file 1 [file ADVS-13-e77138-s001.docx]

**TiO_6_ as a Compensatory Structural Unit in Co-free, High-Ni Layered Oxide for Durable Lithium-Ion Batteries**

*Bixian Ying^a^, Zhenjie Teng^a^, Jun Wang^b^, Honghong Tian^a^, Charlotte von Petersdorff-Campen^a^, Rommel T. Tolla^a^, Iuliia Mikulska^c^, Linus Voigt^a^, Sascha Nowak^a^, Verena Naber^a^, Alexander Schökel^d^, Yuanming Liu^e^, Michael Merz^g,f^, Peter Nagel^g,f^, Stefan Schuppler^g,f^, Katja Frenzel^h^, Adrian Jonas^h^, Lena Mathies^h^, Martin Winter^a,i,*^, Karin Kleiner^a, *^*

*^a^ MEET, Battery Research Center, University of Muenster, Corrensstr. 46, 48149 Muenster, Germany*

*^b^ School of Innovation and Entrepreneurship, Southern University of Science and Technology, 518055 Shenzhen, China*

*^c^ Diamond Light Source Ltd, Harwell Science & Innovation Campus, Didcot, OX11 0DE Oxfordshire, United Kingdom*

*^d^ Deutsches Elektronen-Synchrotron DESY,* *Notkestr. 85, 22607 Hamburg, Germany*

*^e^ Institute of Materials Research, Tsinghua Shenzhen International Graduate School, Tsinghua University, 518055 Shenzhen, China*

*^f^ Institute for Quantum Materials and Technologies, Karlsruhe Institute of Technology, 76021 Karlsruhe, Germany*

*^g^ Karlsruhe Nano Micro Facility (KNMFi), Karlsruhe Institute of Technology (KIT), 76344 Eggenstein-Leopoldshafen, Germany*

*^h^ Physikalisch-Technische Bundesanstalt (PTB), Abbestr. 2-12, 10587 Berlin, Germany*

*^i^ Helmholtz-Institute Münster, IMD-4, Forschungszentrum Jülich GmbH, Corrensstraße 46, 48149 Münster, Germany*

* Corresponding author email address: martin.winter@uni-muenster.de; karin-maria.kleiner@inrs.ca

**General X-ray powder diffraction refinement analysis:**

The Thompson-Cox-Hastings pseudo-Voigt convoluted with axial divergence asymmetry function was used as the normalized peak shape function. The instrumental reflection broadening parameters (U, V, W) were determined separately by using a LaB_6_ NIST standard. These parameters were included in the refinement using an instrumental resolution file (.irf) file. Upon the refinement, the parameters are refined according to the following sequence:

1. Background interpolation and subtraction were used for the refinements. Therefore we have chosen 30 points on the background.
2. The zero shift was refined and left to be free.
3. The operando cell position was adjusted so that the cathode material was in the center of diffraction by comparing the powder diffraction pattern to a capillary measurement performed prior to the experiment. The zero shift was refined once for the OCV measurements and since the position of the operando cell did not change upon cycling, the zero shift was kept constant.
4. The scale factor was refined and was left to be free.
5. The lattice parameters *a*, and *c* were refined according to the space group.
6. The overall Debye-Waller factor (Bov) was refined.
7. The isotropic strain value X and crystallite size value Y were refined.
8. The disorder was refined by introducing two more atoms (Ni in the Li 3b site, and Li in Ni 3a site). The total occupancy was restricted to the stoichiometry.
9. Refinement of Ti in Li 3a site and *TM* 3b site.

Figure S1 Particle size distributions of a) LNO, b) LNTO-1 and c) LNTO-2.

Figure S2 Rietveld refinements of XRD patterns for pristine LNO, LNTO-1, and LNTO-2 materials.

Figure S3 Rietveld refinements of the XRD pattern (measured at synchrotron) for pristine LNTO-2 with different Ti site occupation models: (a) Ti distributed over both *TM* and Li sites, (b) Ti occupying only the *TM* site, and (c) Ti occupying only the Li site. In model (a), the total Ti occupancy was constrained to 0.14 during the refinement. The refinement converged to nearly equal Ti distributions between the *TM* and Li sites, with occupancies of 0.506 and 0.494, respectively. For LNTO-1, a similar mixed-occupancy refinement strategy was also attempted. However, due to the relatively low Ti concentration and the limited scattering contrast between Ni and Ti in X-ray diffraction, the refinement sensitivity for reliably distinguishing Ti occupancy between *TM* and Li sites is limited.

Figure S4 SEM-EDX mappings on the LNTO-1 and LNTO-2 powders.

Figure S5 HRTEM image of LNO, with lattice spacings determined by FFT analysis of the marked regions.

Figure S6 XRD patterns of LNO and LNTO-x samples (x = 0.02, 0.05, 0.07, and 0.14). These patterns were collected using a laboratory X-ray diffractometer (Bruker D8) with mixed Cu Kα radiation (Kα_1_ = 1.5406 Å and Kα_2_ = 1.5418 Å) without a Kα_2_ filter. Therefore, the peak profiles differ slightly from the synchrotron XRD data presented in the manuscript, which were collected at the DESY beamline using monochromatic X-rays.

Figure S7 Rate performance of LNO and LNTO-x samples (x = 0.02, 0.05, 0.07, and 0.14) measured within the voltage window of 3.0–4.4 V. All samples were calcined at 800 °C for consistency and direct comparison.

For consistency, all samples, including the reference LNO, were synthesized using the same calcination temperature of 800 °C, which differs from the LNO sample presented in the main text. As shown in Figure S7, both the cycling stability and rate capability follow the trend: LNTO-2 (0.14) < LNO (0) < LNTO-3 (0.02) < LNTO-4 (0.05) < LNTO-1 (0.07). These results indicate that increasing the Ti content from 0 to 0.07 progressively improves the electrochemical stability and rate performance. However, further increasing the Ti content to 0.14 leads to a slight deterioration in both cycling stability and rate capability.

By contrast, the initial specific capacity and initial coulombic efficiency follow the opposite trend: LNO > LNTO-3 > LNTO-4 > LNTO-1 > LNTO-2, suggesting that excessive Ti incorporation gradually reduces the fraction of electrochemically active Ni species. Nevertheless, except for LNTO-2, the capacity retention after 45 cycles becomes progressively more stable with increasing Ti content, supporting the beneficial structural stabilization effect introduced by moderate Ti substitution.

Based on these results, it can be roughly concluded that x ~ 0.07 provides the best balance between structural stabilization and electrochemical activity in the present system.

Figure S8 SEM images of (a) LNO electrode and (b) LNTO-1 electrode after 150 cycles. The red circles mark the particle cracks.

Figure S9 Rietveld refinements of XRD patterns for fully charged LNO and LNTO-1 materials. Some regions were excluded due to the presence of the Al phase from the current collector.

Figure S10 SXPD patterns of (a) LNTO-1 and (b) LNO during first cycling.

Figure S11 Waterfall plots of the phase transition diagram of LNO upon charging

Figure S12 *In situ* XANES technique reveals oxidation state changes during initial cycling. XANES data for pristine samples and reference TiO_2_: (a) Ni K edge, (d) Ti K edge. XANES data for cycled samples: Ni K edge on (b) LNTO-1, and (c) LNO, and (e) Ti K edge on LNTO-1. Ni K edge spectra were measured *in situ*, while Ti K edge spectra were measured *ex situ*. XANES spectra shown on (b), (c) and (e) figure panes are shifted vertically for clarity.

Figure S13 EXAFS Ni K-edge fitting results of LNO at OCV, charged to 4.4 V, and discharged to 3.0 V. Fitting was performed using Artemis software. The blue dashed boxes indicate the fitting regions. Panels (a), (c), and (e) show fits using two coordination shells: Ni–O and Ni–*TM*. Panels (b), (d), and (f) use three shells: Ni–O1, Ni–O2, and Ni–*TM*, with coordination numbers of 4 and 2 for Ni–O1 and Ni–O2, respectively. Black circles highlight regions where the fitting is less accurate. Overall, the LNO sample is better modeled using three shells (Ni–O1, Ni-O2 and Ni–*TM*), suggesting the existence of Jahn–Teller distortion at the Ni K-edge.

Figure S14 EXAFS Ni K-edge fitting results of LNTO-1 at OCV, charged to 4.4 V, and discharged to 3.0 V. Fitting was performed using Artemis software. The blue dashed boxes indicate the fitting regions. Panels (a), (c), and (e) show fits using two coordination shells: Ni–O and Ni–*TM*. Panels (b), (d), and (f) use three shells: Ni–O1, Ni–O2, and Ni–*TM*, with coordination numbers of 4 and 2 for Ni–O1 and Ni–O2, respectively. Black circles highlight regions where the fitting is less accurate. Overall, the LNTO-1 sample is better modelled using only two shells (Ni–O and Ni–*TM*), suggesting the absence of Jahn–Teller distortion at the Ni K-edge.

Figure S15 Wavelet-transformed Ni K edge EXAFS of LNO at OCV, charged to 4.4 V, and discharged to 3.0 V.

Figure S16 Schematic diagram of edge sharing characteristics between TiO_6_ and neighboring NiO_6_ or LiO_6_ octahedra in a $R\bar{3}m$ unit cell. a) Ti occupies the Ni site (3a), b) Ti occupies the Li site (3b).

**Independent Verification of SOC-Dependent NEXAFS Data for LNTO-1**

This section presents the SOC-dependent NEXAFS spectra of the LNTO-1 sample, independently reproduced by the Physikalisch-Technische Bundesanstalt (PTB) at the U49 PGM beamline located in BESSY II[1]. This data is included to provide a reliable verification of the NEXAFS measurements presented and discussed in the main part of this paper. The characteristic spectral features and their evolution as a function of SOC are consistently observed in both data sets. This independent verification underlines the robustness of the experimental results and further supports the proposed stabilisation mechanism of Ti substitution in the LiNiO_2_ cathode material.

Figure S17 NEXAFS data of cycled LNTO-1 at the O K (a), Ni L (b), and Ti L (c) edges, measured in fluorescence yield mode, are shown for pristine, 0%, 50%, 75%, and 100% SOCs in the discharged state. These measurements were conducted at PTB in Berlin as an independent verification for Figure 5.

Figure S18 NEXAFS data of cycled samples at the 1^st^ cycle, and the 100^th^ cycle on O K (a), Ni L (b), and Ti L (c) edges in fluorescence yield. Note that the Ti L-edge spectra of TiO_2_ and LNTO-1 cannot be normalized using the same procedure because TiO_2_ exhibits a pronounced edge jump, whereas the Ti signal in LNTO-1 is much weaker due to the significantly lower Ti concentration, resulting in almost no obvious edge jump in the FY spectra. Therefore, the comparison is intended to provide qualitative rather than quantitative information.

Figure S19 NEXAFS data of pristine and cycled samples (LNO and LNTO-1) the 1^st^ cycle, and the 100^th^ cycle on O K (a), Ni L (b), and Ti L (c) edges in total electron yield.

Note that the overall Ti L-edge intensity decreases after 100 cycles, accompanied by slight changes in the spectral shape. This attenuation may be associated with the formation of a thicker cathode electrolyte interphase (CEI) layer on the particle surface, which can dampen the X-ray signal, particularly due to the presence of fluorine-containing surface species [2]. In addition, possible changes in the local Ti–O coordination environment during cycling may also contribute to the observed spectral evolution. However, based on the present NEXAFS and XPS depth profiling in Figure S20 results alone, it is difficult to unambiguously distinguish the individual contributions from CEI formation, local structural disorder, or possible amorphization effects.

Figure S20 XPS depth profiling of LNTO-1 after 100 cycles: a) Li 1s, b) Ti 2p, c) O 1s, and Ni 2p edges. Different durations of seconds correspond to varying etching times, enabling the probing of samples at different thicknesses.

As shown in Figure S20a and Figure S20d, the Ni 2p3/2 and Ni 2p1/2 spectra at the surface region (etching time = 0 s) appear relatively broad and less well resolved due to the presence of surface species and CEI components. With increasing etching time, both the peak intensity and spectral resolution gradually improve, indicating the progressive removal of surface impurities and exposure of the underlying bulk layered oxide framework. The O 1s spectra in Figure S20c further support this interpretation. At the surface region (etching time = 0 s), a dominant carbonate-related peak is observed, which can be assigned to Li_2_CO_3_ or other carbonate-containing surface species originating from CEI formation. As the etching time increases, the carbonate-related contribution gradually decreases, while the lattice oxygen peak becomes increasingly pronounced. After approximately 300 s of etching, the lattice oxygen peak intensity becomes nearly stable, suggesting that most surface species have been removed and that the bulk region is predominantly being probed. Unfortunately, the Ti concentration is below the detection limit in the present measurements, preventing a direct evaluation of possible Ti dissolution through the Ti 2p signal.

Tables:

Table S1 ICP-OES results of LNO, LNTO-1, and LNTO-2, showing the measured total elemental compositions. During the microwave digestion process, LNTO-1 and LNTO-2 initially exhibited slight turbidity and greenish residues, which gradually disappeared after standing for a short period, resulting in clear solutions suitable for ICP-OES measurements.

| Samples | Li | Ni | Ti |
| --- | --- | --- | --- |
| LNO | 1.00 | 1.00 |  |
| LNTO-1 | 1.00 | 0.93 | 0.07 |
| LNTO-2 | 1.00 | 0.86 | 0.14 |

To give the reader a better understanding of how the refinement of the data was performed, the following tables give parameters used and obtained upon data processing. R_Bragg_ values are given for the most important refinements. They reflect the difference between the observed and calculated structure factors normalized to the observed values, multiplied by their multiplicity. Note that Rp values are not actively included to minimize mismatches in the refinement. The table also includes the estimated standard deviations of lattice parameters obtained from the Rietveld refinement analysis. These standard deviations have been multiplied by correlated residuals[3, 4].

Table S2 Crystallographic parameters of LNO.

| LNO Cell Parameters[5] | | | | | |
| --- | --- | --- | --- | --- | --- |
| Space group: *R-3mH*, *a* = *b* = 2.8645 Å, *c* = 14.161 Å, gamma = 120°, *V* = 100.63 Å^3^, *Z* = 3 | | | | | |
| Atomic positions | | | | | |
| Name | site | x | y | z | Fract. |
| Li1 | 3b | 0.00 | 0.00 | 0.50 | 0.978 |
| Ni1 | 3b | 0.00 | 0.00 | 0.50 | 0.022 |
| Li2 | 3a | 0.00 | 0.00 | 0.00 | 0.022 |
| Ni2 | 3a | 0.00 | 0.00 | 0.00 | 0.978 |
| O1 | 6c | 0.00 | 0.00 | 0.2585 | 1.00 |

Table S3 Crystallographic parameters of LNTO-1.

| LiNTO-1 Cell Parameters | | | | | |
| --- | --- | --- | --- | --- | --- |
| Space group: *R-3mH*, *a* = *b* = 2.87927 Å, *c* = 14.22812 Å, gamma = 120°, V = 102. Å^3^, *Z* = 3 | | | | | |
| Atomic positions | | | | | |
| Name | site | x | y | z | Fract. |
| Li1 | 3b | 0.00 | 0.00 | 0.50 | 0.951 |
| Ni1 | 3b | 0.00 | 0.00 | 0.50 | 0.014 |
| Ti1 | 3b | 0.00 | 0.00 | 0.50 | 0.035 |
| Li2 | 3a | 0.00 | 0.00 | 0.00 | 0.014 |
| Ni2 | 3a | 0.00 | 0.00 | 0.00 | 0.951 |
| Ti2 | 3a | 0.00 | 0.00 | 0.00 | 0.035 |
| O1 | 6c | 0.00 | 0.00 | 0.2585 | 1.00 |

Table S4 Crystallographic parameters of LNTO-2.

| LiNTO-2 Cell Parameters | | | | | |
| --- | --- | --- | --- | --- | --- |
| Space group: *R-3mH*, *a* = *b* = 2.88349 Å, *c* = 14.25909 Å, gamma = 120°, V = 102. Å^3^, *Z* = 3 | | | | | |
| Atomic positions | | | | | |
| Name | site | x | y | z | Fract. |
| Li1 | 3b | 0.00 | 0.00 | 0.50 | 0.908 |
| Ni1 | 3b | 0.00 | 0.00 | 0.50 | 0.022 |
| Ti1 | 3b | 0.00 | 0.00 | 0.50 | 0.070 |
| Li2 | 3a | 0.00 | 0.00 | 0.00 | 0.022 |
| Ni2 | 3a | 0.00 | 0.00 | 0.00 | 0.908 |
| Ti2 | 3a | 0.00 | 0.00 | 0.00 | 0.070 |
| O1 | 6c | 0.00 | 0.00 | 0.2585 | 1.00 |

Table S5 Pristine materials refinement analysis results.

| sample | *a* = *b* lattice parameter/ Å | *c* lattice parameter/ Å | Uni cell volume/ Å^3^ |
| --- | --- | --- | --- |
| LNO | 2.87551(7) | 14.19020(50) | 101.613(1) |
| LNTO-1 | 2.87927(5) | 14.22812(33) | 102.151(1) |
| LNTO-2 | 2.88349(7) | 14.25909(53) | 102.674(1) |

Table S6 Comparison of energy positions of Ni K-edge XANES features. The energy values of main edge correspond to the maximum of the derivative of the normalized absorption coefficient, µ(E) in Ni K edge.

|  | Pristine | Full charge | Full discharge |
| --- | --- | --- | --- |
| Samples | Main edge/ eV | Main edge/ eV | Main edge/ eV |
| LNO | 8347.81 | 8351.14 | 8347.90 |
| LNTO-1 | 8347.87 | 8350.34 | 8347.90 |

Table S7 LNO Ni K edge EXAFS fitted coordination numbers, distances and distortion factor (σ2) of the three-paths model.

| LNO Ni K | Shell | N | R(Å) | σ2(Å^2^) | R-factor |
| --- | --- | --- | --- | --- | --- |
| OCV | Ni-O1 | 4 | 1.96422±0.00709 | 0.01677±0.00319 | 0.00252 |
|  | Ni-O2 | 2 | 2.25744±0.00539 | 0.01344±0.00010 |  |
|  | Ni-*TM* | 6 | 2.86362±0.00725 | 0.00525±0.00066 |  |
| Charge-4.4V | Ni-O1 | 2 | 1.85252±0.08021 | 0.00100±0.00050 | 0.00370 |
|  | Ni-O2 | 4 | 1.91724±0.06750 | 0.00636±0.00130 |  |
|  | Ni-*TM* | 6 | 2.75198±0.00867 | 0.00565±0.00060 |  |
| Discharge-3.0V | Ni-O1 | 4 | 1.88669±0.00952 | 0.00114±0.00050 | 0.00319 |
|  | Ni-O2 | 2 | 2.04820±0.00823 | 0.00273±0.00069 |  |
|  | Ni-*TM* | 6 | 2.87187±0.00773 | 0.00657±0.00134 |  |

N= coordination number; R= distance between absorber and backscatter atoms; R-factor- the goodness of fitting

Table S8 LNTO-1 Ni K edge EXAFS fitted coordination numbers, distances and distortion factor (σ2) of the two-paths model.

| LNTO-1 Ni K | Shell | N | R(Å) | σ2(Å^2^) | R-factor |
| --- | --- | --- | --- | --- | --- |
| OCV | Ni-O | 6 | 1.94321±0.02766 | 0.00816±0.00491 | 0.00064 |
|  | Ni-*TM* | 6 | 2.88635±0.02602 | 0.00526±0.00246 |  |
| Charge-4.4V | Ni-O | 6 | 1.88659±0.01320 | 0.00464±0.00264 | 0.00268 |
|  | Ni-*TM* | 6 | 2.82581±0.00749 | 0.00521±0.00133 |  |
| Discharge-3.0V | Ni-O | 6 | 1.90734±0.01883 | 0.00614±0.00298 | 0.00280 |
|  | Ni-*TM* | 6 | 2.85336±0.02917 | 0.00573±0.00164 |  |

Table S9 LNTO-1 Ti K edge EXAFS fitted coordination numbers, distances and distortion factor (σ2) of the three-paths model.

| LNTO-1 Ti K | Shell | N | R(Å) | σ2(Å^2^) | R-factor |
| --- | --- | --- | --- | --- | --- |
| OCV | Ti-O1 | 4.93 | 1.84358±0.07257 | 0.002782±0.00085 | 0.00440 |
|  | Ti-O2 | 1.07 | 1.99866±0.08039 | 0.002576±0.00063 |  |
|  | Ti-*TM* | 6 | 2.87355±0.02191 | 0.00376±0.00099 |  |
| Charge-4.4 V | Ti-O1 | 3.71 | 1.82020±0.03345 | 0.01201±0.00374 | 0.00427 |
|  | Ti-O2 | 1.29 | 1.98942±0.01969 | 0.01114±0.00470 |  |
|  | Ti-*TM* | 6 | 2.88864±0.01904 | 0.01309±0.00406 |  |
| Discharge-3.0 V | Ti-O1 | 4.99 | 1.85035±0.00935 | 0.00209±0.00099 | 0.01522 |
|  | Ti-O2 | 1.01 | 1.99578±0.07478 | 0.001978±0.00078 |  |
|  | Ti-*TM* | 6 | 2.88989±0.08989 | 0.004438±0.00123 |  |

The sum of the coordination numbers for Ti–O1 and Ti–O2 should be 6, consistent with the expected octahedral coordination of Ti.

References

1.	Scholze, F., et al. *New PTB beamlines for high-accuracy EUV reflectometry at BESSY II*. in *Soft X-Ray and EUV Imaging Systems*. 2000. SPIE.

2.	Kleiner, K., et al., “*On the Origin of Reversible and Irreversible Reactions in LiNixCo (1− x)/2Mn (1− x)/2O2*,” Journal of The Electrochemical Society(2021): **168**(12): p. 120533.

3.	Bérar, J.-F. and P. Lelann, “*ESD's and estimated probable error obtained in Rietveld refinements with local correlations*,” Journal of applied crystallography(1991): **24**(1): p. 1-5.

4.	Berar, J., “*Data optimization and propagation of errors in powder diffraction,“Acc*,” Pow. Diff II”, NIST sp. Pub(1992): **846**: p. 63.

5.	Pouillerie, C., E. Suard, and C. Delmas, “*Structural characterization of Li1− z− xNi1+ zO2 by neutron diffraction*,” Journal of Solid State Chemistry(2001): **158**(2): p. 187-197.
